# Scavenger community structure along an environmental gradient from boreal forest to alpine tundra in Scandinavia

**DOI:** 10.1002/ece3.6834

**Published:** 2020-09-25

**Authors:** Gjermund Gomo, Lars Rød‐Eriksen, Harry P. Andreassen, Jenny Mattisson, Morten Odden, Olivier Devineau, Nina E. Eide

**Affiliations:** ^1^ Faculty of Applied Ecology, Agricultural Sciences and Biotechnology (Fac. Appl. Ecol.) Inland Norway University of Applied Sciences (INN) Koppang Norway; ^2^ Norwegian Institute for Nature Research (NINA) Trondheim Norway; ^3^ Department of Biology Centre for Biodiversity Dynamics (CBD) Norwegian University of Science and Technology (NTNU) Trondheim Norway

**Keywords:** alpine tundra, bait, boreal forest, camera traps, community, scavengers

## Abstract

Scavengers can have strong impacts on food webs, and awareness of their role in ecosystems has increased during the last decades. In our study, we used baited camera traps to quantify the structure of the winter scavenger community in central Scandinavia across a forest–alpine continuum and assess how climatic conditions affected spatial patterns of species occurrences at baits. Canonical correspondence analysis revealed that the main habitat type (forest or alpine tundra) and snow depth was main determinants of the community structure. According to a joint species distribution model within the HMSC framework, species richness tended to be higher in forest than in alpine tundra habitat, but was only weakly associated with temperature and snow depth. However, we observed stronger and more diverse impacts of these covariates on individual species. Occurrence at baits by habitat generalists (red fox, golden eagle, and common raven) typically increased at low temperatures and high snow depth, probably due to increased energetic demands and lower abundance of natural prey in harsh winter conditions. On the contrary, occurrence at baits by forest specialists (e.g., Eurasian jay) tended to decrease in deep snow, which is possibly a consequence of reduced bait detectability and accessibility. In general, the influence of environmental covariates on species richness and occurrence at baits was lower in alpine tundra than in forests, and habitat generalists dominated the scavenger communities in both forest and alpine tundra. Following forecasted climate change, altered environmental conditions are likely to cause range expansion of boreal species and range contraction of typical alpine species such as the arctic fox. Our results suggest that altered snow conditions will possibly be a main driver of changes in species community structure.

## Graphical abstract

In our study, we used baited camera traps to quantify the structure of the winter scavenger community in central Scandinavia across a forest–alpine continuum and assess how climatic conditions affected spatial patterns of species occurrences. Following forecasted climate change, altered environmental conditions is likely to cause range expansion of boreal species and range contraction of typical alpine species. Our results suggest that altered snow conditions will be a main driver of change.

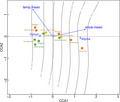

## 1 INTRODUCTION

Scavengers are an important component of ecosystems due to their effect on nutrient cycling, stabilizing food webs, and disease transmission (Mateo‐Tomás et al., 2017). Interest in scavenging has increased during the last decade, and recent studies have highlighted that scavengers may impact food webs in more intricate ways than only consumption of carrion (Wilson & Wolkovich, 2011). The traditional focus on obligate scavengers and bottom‐up processes have been broadened to include facultative scavengers that potentially have a strong impact on coexisting prey and predator species through both direct and indirect interactions (Pereira et al., 2014; Wilson & Wolkovich, 2011).

Large scale patterns in scavenger guild structure are driven by species' physiological capability, landscape productivity, climatic constraints, as well as inter‐ and intraspecific competition (Elmhagen et al., 2015; Mateo‐Tomás et al., 2015; Moleón et al., 2014). As a consequence, different large scale habitats (e.g., forest, tundra, grassland) likely host rather different scavenger guilds, despite some scavengers occurring across various habitats (Arrondo et al., 2019; Pardo‐Barquín et al., 2019). A recent review of global patterns in vertebrate scavenger distribution concluded that the degree of human impact was a main predictor of richness in the scavenging guild (Sebastián‐González et al., 2019). Along a human footprint index overall species richness was lowest at high index values and highest at medium values. Scavenger richness was highest at carrion of medium size and in the winter season, while average temperature and rainfall had no effect on scavenger richness.

In the boreal forest and alpine tundra habitat of Scandinavia, the vertebrate scavenger guild consists of opportunistic facultative scavengers ranging from small sized terrestrial mammals and birds to large carnivores and raptors (Gomo et al., 2017; Henden et al., 2014; Killengreen et al., 2012). Harsh winter conditions in the alpine tundra habitats may function as a barrier for boreal forest species. However, climate change affects both temperature and precipitation, and hence overall primary productivity, which opens the possibility for northwards expansion for species limited by these factors (Elmhagen et al., 2015; Gomo et al., 2017). Climate change at northern latitudes also impacts the cover, depth, and structure of snow, which, for example, is likely to influence the predators’ access to rodents and other food sources (Halpin & Bissonette, 1988; Willebrand et al., 2017). Deep snow can also restrict locomotion and area use in mammals (Pozzanghera et al., 2016).

Many scavengers utilize carrion resources in northern areas during winter, thus potentially strengthening food web interactions between species that otherwise have weak connections in these ecosystems (Moleón et al., 2014). For instance, carrion consumption by red fox (*Vulpes vulpes*), an important generalist predator, increase during periods of low natural prey availability (Jędrzejewski & Jędrzejewska, 1992; Killengreen et al., 2011; Needham et al., 2014). The documented increase in ungulate populations during the last century have generated a large amount of ungulate carrion available for scavenging species, including gut piles left in the field by hunters (Hagen, 2014; Selås & Vik, 2006; Wikenros et al., 2013). The expansion of boreal associated species into alpine and arctic tundra habitats can be facilitated by access to carrion (Killengreen et al., 2012; Sokolov et al., 2016). For example, high abundance of carrion from semi‐domesticated reindeer (*Rangiferus tarandus*) has been linked to high occupancy of scavenging species including corvids, eagles, and red fox on the arctic tundra in northern Scandinavia (Henden et al., 2014).

This study aims to quantify the structure of the winter scavenger community across a forest–alpine gradient in central Scandinavia, with a special focus on the underlying drivers of the occurrence at baits by different scavenging species with respect to climatic conditions and habitat types. We did this by placing baited camera traps along the forest–alpine gradient. In general, species richness at baits is expected to be higher in forest than in alpine tundra habitats, but snow depth and temperatures can influence species‐specific occurrence at baits within and between habitat types, resulting in complex structures of the scavenger guild.

Some scavenging species are likely very restricted to their preferred habitats, while generalists are typically not so selective. We predict that the scavenger guild structure in large will be clustered into habitat specialists and habitat generalists. Scavenger species could also be restricted by climatic conditions. We predict that both temperature and snow depth are important factors structuring the scavenger guild by limiting their distribution and affecting their activity patterns when present. Snow can also restrict species utilization of food resources such as, for example, carrion. We predict that snow depth will have the greatest impact on species utilizing food sources or prey which might be covered in snow, for example, small rodents or plants. Since birds mostly locate food by eyesight and have limited ability to dig through deep snow, we predict that snow depth might affect bird scavenging more than mammal scavenging.

## 2 MATERIALS AND METHODS

### 2.1 Study area

**FIGURE 1:**
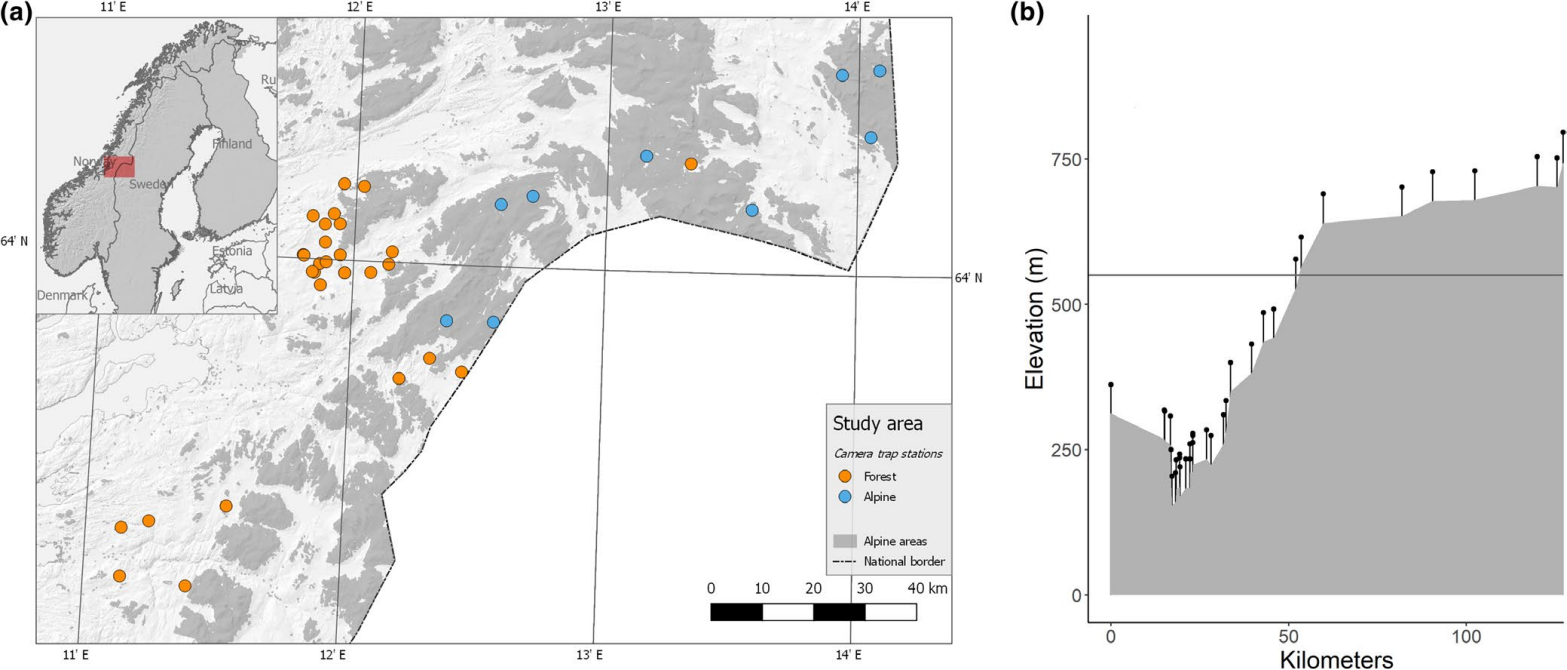
(a) Spatial distribution of study sites in central Norway. Circles show the placement of camera traps in boreal forest habitats (orange) and alpine tundra habitats (blue). Gray areas represent alpine tundra habitat. (b) Distribution of camera trap sites (pinned dots) along an elevational gradient on a longitudinal axis from west to east. The horizontal line illustrates the forest line

Our study was conducted between January and April in the areas of Lierne, Blåfjella and Skjækerfjella in central Norway from 2012 to 2014 (Figure 1a). The area covers an elevational gradient ranging from 90 to 850 m.a.s.l., with the forest line at approximately 560 m.a.s.l. (Figure 1b). Alpine tundra habitats are dominated by dwarf birch (*Betula nana*) and shrubs of willow (*Salix* sp.), whereas forested habitats are dominated by pine (*Pinus sylvestris*), spruce (*Picea abies*), and mountain birch (*Betula pubescens*) (Moen, 1998). Semi‐domesticated reindeer have perennial pastures within the region, including calving areas within or bordering our study area. Wild ungulates are mainly moose and roe deer. Carrion from ungulates provide an estimated biomass of 29.1 kg/km^2^ in boreal forest and 3.6 kg/km^2^ in alpine tundra areas during the cold season (November to April) (Hagen, 2014).

### 2.2 Study design and field sampling

Camera traps (Reconyx Hyperfire Professional PC 800 and PC 900, Reconyx Inc.) were set up at 38 locations (29 in forest and 9 in alpine tundra habitat) for a total of 65 (42 in forest and 23 in alpine tundra habitat) bait sessions, that is, camera site per year, and 1,253 monitoring days between January and April from 2012 to 2014. Baits in alpine tundra habitat were monitored at the same site all three years (but 4 bait sessions were excluded due to failure to observe any species) while the location of baits in the forest changed between the years creating the high number of locations. Bait sessions started when bait was deployed and ended when bait was depleted. The average duration of a bait session was 19 ± 4 days (mean ± 2*SE*) in forest and 20 ± 5 days in alpine tundra habitats, ranging from 4 to 62 days. Bait consisted of frozen blocks of discarded reindeer meat, fat, and connective tissue weighing between 10 and 20 kg and measuring approximately 60 × 40 × 15 cm. To ensure that the bait was not removed immediately by large scavengers and to keep the bait frozen, the bait was buried in the snow in a vertical position such that only 5 cm of the top was visible. Cameras were placed 4–6 m from the baits at approximately 1–1.5 m above the ground and tilted slightly downwards toward the bait. Camera traps were programmed to take a picture every 10 min to increase capture probability of elusive species (c.f. Hamel et al., 2013). All pictures were examined and the number of individuals of each species in each picture was recorded. To reduce sampling bias, only images where the bait was present (i.e., not consumed) and thus acting as an attractant to animals were included in the analyses (Figures S7a–c). The time from baits were placed out to the first picture of any scavenger was similar between forest ($\bar{x}$ = 5.7 days ± 2.10 *SE*) and alpine tundra ($\bar{x}$ = 7.0 days ± 3.87 *SE*) habitats.

### 2.3 Statistical analyses

We used canonical correspondence analysis (CCA) (Ter Braak, 1986) to structure the species community in relation to environmental variables. We included presence/absence data for 13 species, aggregated per camera site within each year, resulting in a total of 61 bait sessions (i.e., site‐years), four sites with no observations were removed to reduce noise in the models. Small mustelids (*Mustela erminea*, *Mustela nivalis*), goshawk (*Accipiter gentilis*), and rough‐legged buzzard (*Buteo lagopus*) were excluded from the CCA analysis due to too few site observations. Habitat, that is, alpine tundra (above forest line) or forest (below forest line), was included as a categorical variable. Daily means of temperature and snow depth were extracted from interpolated maps (NMI, 2019) with a spatial resolution of 1 × 1 km pixels and calculated as an average within a 1.5 km buffer (~7 km^2^) around each camera site for each bait session. The size of the buffer was selected to average over multiple pixels encompassing a camera trap site, as a site could potentially be located at the fringes of a singular 1 × 1 km pixel which might not be representable for the overall snow depth or temperature at the site. Mean site temperature (± 2*SE*) over all study sites and years was −0.46°C (± 0.72) at forested and −4.02°C (± 1.37) at alpine tundra sites, whereas mean snow depth was 32 cm (± 9.9) at forested and 107.7 cm (± 9.9) at alpine tundra sites.

We mostly followed the procedure of CCA modeling described in Ter Braak and Verdonschot (1995) to explore the effects of the included environmental variables on the scavenger community structure, and created a set of candidate models from the three environmental variables. The models were compared through an analysis of variance (ANOVA). CCA models were constructed and visualized using the package “vegan” in R version 3.6.1 (Oksanen et al., 2018; R‐Core‐Team, 2019).

We utilized a joint species distribution model (JSDM) within the hierarchical modeling of species communities framework (Ovaskainen et al., 2017) using the package “Hmsc” (Tikhonov et al., 2019) in R to model average site‐level species richness and species‐specific distribution along environmental gradients. This framework was preferred over occupancy models due to an unbalanced study design where most camera trap sites within forest habitats were active in only 1 or 2 years, thus reducing the number of repeated measures over years and revealing spurious occupancy estimates. Furthermore, occupancy models have been found to overestimate the probability of area use for rare and highly mobile species captured by camera traps (e.g., Neilson et al., 2018), that is, >50% of the species identified in our study. Although we recognize that the Hmsc framework currently does not account for imperfect detection, we considered this framework more robust when dealing with unbalanced species occurrence data.

The Hmsc framework was utilized in a basic capacity form as a multivariate linear mixed model with a binomial (presence/absence) distribution with a probit link function. The basic model was defined as:

$$y_{\mathrm{ij}}=\alpha_{j}+\beta_{1j}x_{1i}+\beta_{2j}x_{2i}+\beta_{3j}x_{3i}+\varepsilon_{\mathrm{ij}}^{S}$$

where *y* is a matrix of presence/absence (1/0) data corresponding to site/years (bait session *i*) and species (*j*), *α* and *β* are the true intercept and slope parameters for covariates, and *x* the three main covariates included (habitat, snow depth, and temperature). The *ε* parameter represents here the random effect at the site level (*S*; Camera trap ID) to account for temporal autocorrelation between sites with repeated measures (i.e., >1 year of sampling). We included all observed species to estimate species richness, retaining the 61 bait sessions used in the CCA analysis. We used the same environmental variables as in the CCA analysis; habitat (forested/alpine tundra), snow depth, and temperature, as predictors of species richness and occurrence at baits. The interaction between habitat and each environmental variable was included in the models to assess species‐specific responses to environmental gradients within each habitat. The posterior distribution was sampled using MCMC with 7,500 samples over 2 chains, a thinning of 5 and burn‐in of 2,500 samples. MCMC convergence was evaluated using trace plots of *β* parameters, by comparing effective sample sizes of each parameter to the total number of samples, and by potential scale reduction factors (Gelman & Rubin, 1992; Plummer et al., 2006). The trace plots revealed similar patterns for both chains and no sign of autocorrelation. Most potential scale reduction factors were centered around 1.00 (i.e., the number of chains gave consistent results), however, the effective sample size was generally lower than the maximum posterior samples drawn (2,000), which is not uncommon with non‐normally distributed data (Tikhonov et al., 2019) (Figure S1). The explanatory power of the model for each species was validated using Tjur's *D* (coefficient of discrimination; Tjur, 2009) which showed quite low explanatory power of the model on species probability of occurrence at baits (*D* < 0.2; Figure S2a). However, the predictive power of the model, evaluated from a fivefold cross validation, was equivalent to the explanatory power, indicating relatively good model fit (Figure S2b).

## 3 RESULTS

We included 217,951 photos in the analysis, from which we observed 15 scavenging species at the bait stations during the study period, including nine bird species and seven mammalian species (Table 1). Seven species were found only in forest, 2 only in alpine tundra habitat, whereas 6 species were found in both habitats.

**TABLE 1:** Identified scavenging species from baited camera traps and their daily visiting rate as a percentage of total camera trap days within forested and alpine tundra habitats (*Trap days*)

Species	Linnaeus, 1758	Trap days (%)			Sites (*n* = 61)		
		Forest	Alpine	All	Forest	Alpine	Photos
Eurasian jay (*ej*)	*Garrulus glandarius*	25.1	0	15.7	23	0	2,523
Siberian jay (*sj*)	*Perisoreus infaustus*	5.9	0	3.7	7	0	270
Magpie (*mp*)	*Pica pica*	2.9	0	1.8	12	0	377
Goshawk (*gh*)	*Accipiter gentilis*	1.3	0	0.8	2	0	133
Small mustelids (*mus*)	*M. erminea, M. nivalis*	0.3	0	0.2	2	0	4
Badger (*bg*)	*Meles meles*	1.9	0	1.2	3	0	28
Pine marten (*pm*)	*Martes martes*	7.8	0	4.9	11	0	162
White‐tailed eagle (*wte*)	*Haliaeetus albicilla*	0.9	0.2	0.6	7	1	52
Hooded crow (*hc*)	*Corvus cornix*	9.2	2.1	6.5	21	4	4,357
Golden eagle (*ge*)	*Aquila chrysaetos*	8.9	9.0	8.9	23	17	1,285
Common raven (*cr*)	*Corvus corax*	12.5	25.8	17.5	27	18	5,903
Red fox (*rf*)	*Vulpes vulpes*	17.1	22.6	19.2	31	19	1897
Wolverine (*wo*)	*Gulo gulo*	1.3	3.2	2.0	4	7	133
Rough‐legged buzzard (*rlb*)	*Buteo lagopus*	0	0.2	0.1	0	1	1
Arctic fox (*af*)	*Vulpes lagopus*	0	0.9	0.3	0	3	16

Note

Sites refer to the pooled number of active sites over all study years with observations of the species. Photos are the total number of images recorded of the species over all camera sites and years.

### 3.1 Scavenger community structure

**FIGURE 2:**
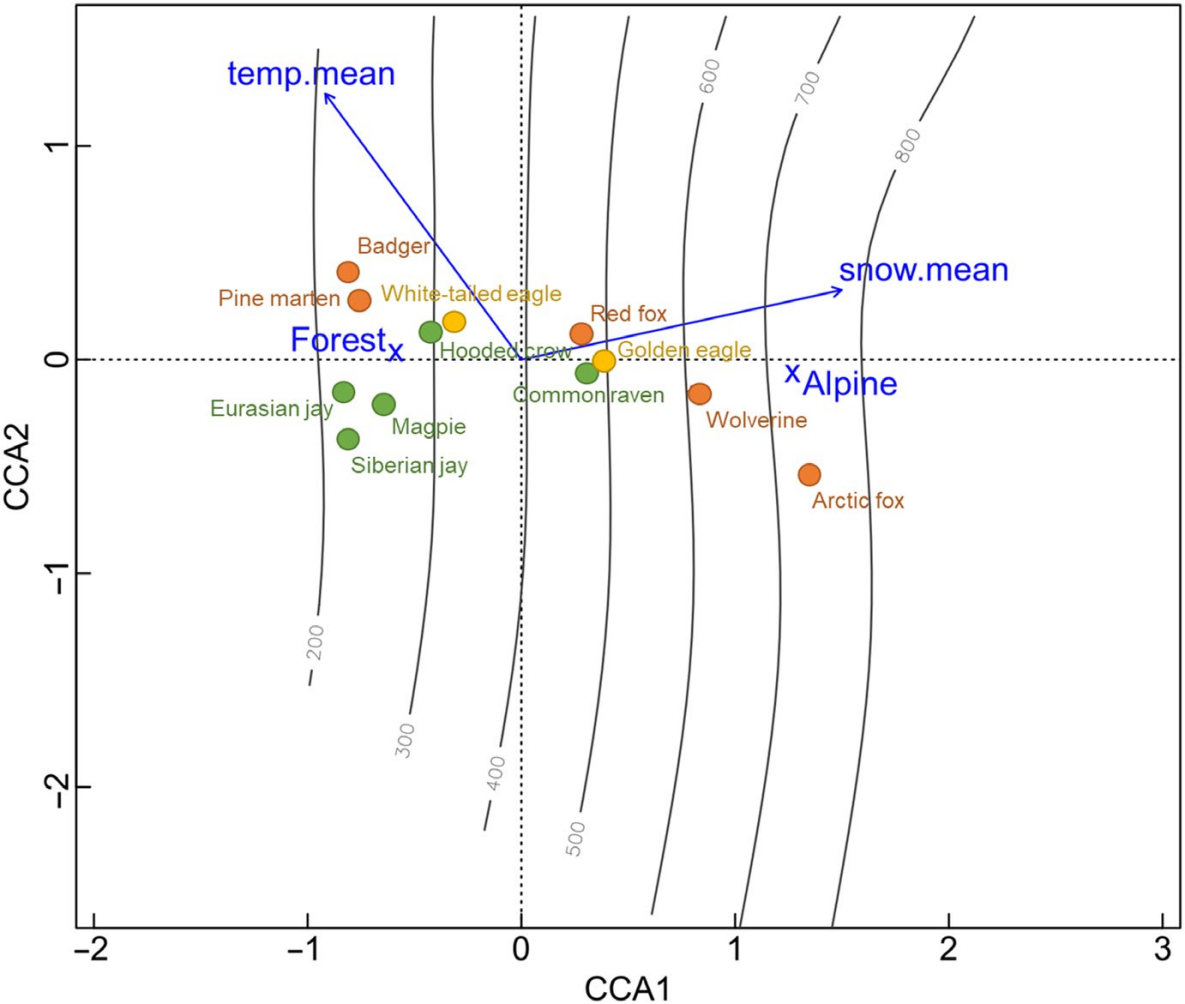
Results from a canonical correspondence analysis (CCA) showing habitat preference of the species structured along environmental gradients, additionally illustrated by altitudinal isoclines (100‐m intervals). Blue arrows, crosses, and text represents the environmental gradients, where arrows show the direction of continuous environmental variables (temperature and snow depth). Species distribution within the environmental space is illustrated by colored circles, where green circles = corvids, yellow = raptors, and red = mammals

**FIGURE 3:**
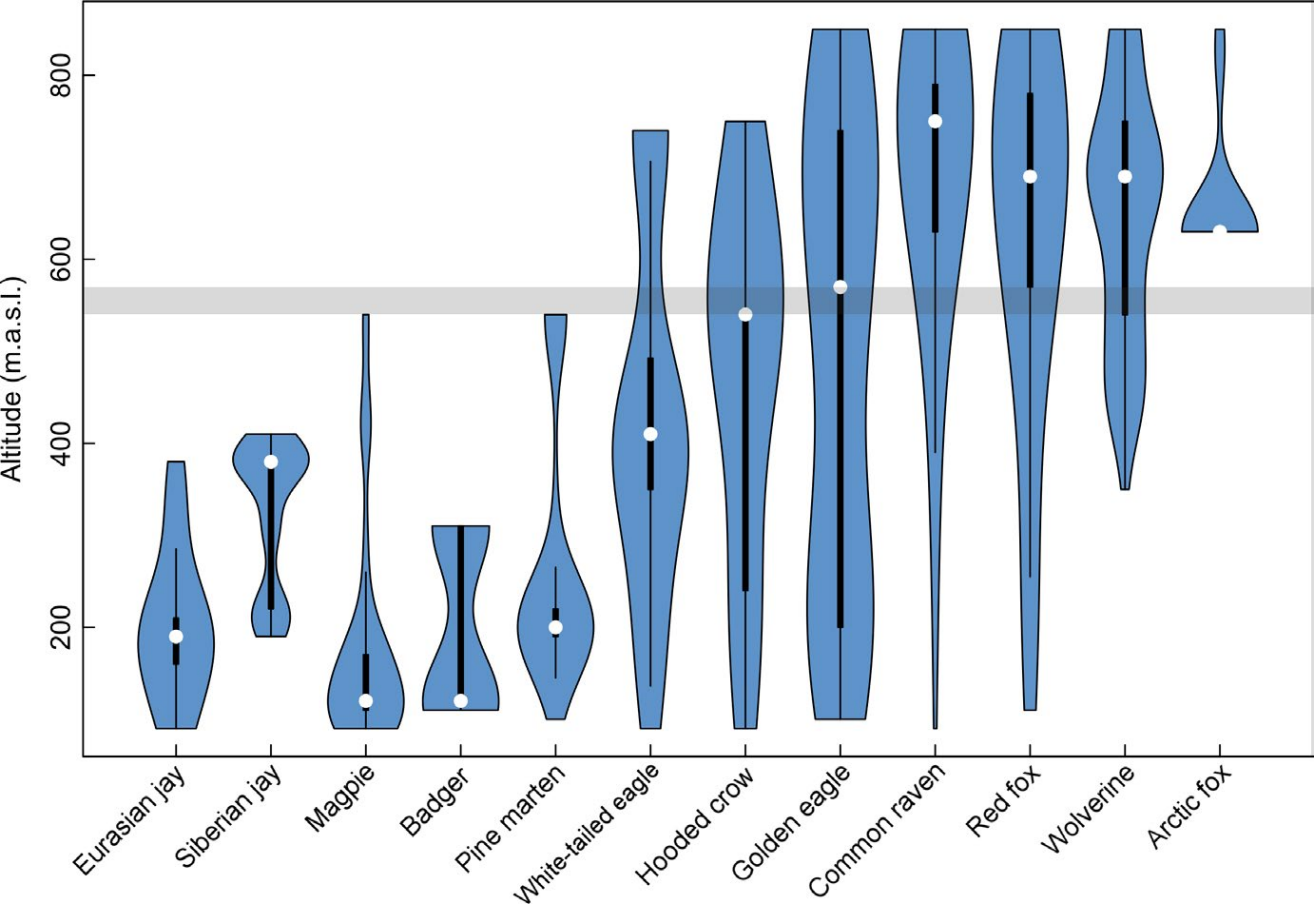
The distribution of observed scavenger species along an altitudinal gradient, based on the number of photos of each species. Thickness of vertical columns indicates frequency of observations (thicker = more observations). White dots represent the median number of observations. Bold vertical lines indicate variability within the lower and upper quartile, whereas thin vertical lines indicate variability between minimum and maximum values. The shaded gray area represents the gap between forested (90–540 m.a.s.l.) and alpine tundra (570–850 m.a.s.l.) habitat classifications. Small mustelids, goshawk, and rough‐legged buzzard are not included in the plot due to too few site observations

The structure of the scavenger guild along environmental gradients, as determined from the exploratory CCA analysis, was largely explained by habitat and mean snow depth (Figure 2; Table S2). Axis 1 (CCA1) explained 91.1% (eigenvalue = 0.27) of the constrained variance, with relatively strong effects of habitat and snow depth. Axis 2 (CCA2) explained 6.0% (eigenvalue = 0.02) of the constrained inertia and was not statistically significant (Table S1). Overall, the included variables explained 18.4% of the variance in the model. The three smallest corvid species (Eurasian jay (*Garrulus glandarius*), Siberian jay (*Perisoreus infaustus*), magpie (*Pica pica*), badger (*Meles meles*), and pine marten (*Martes martes*) occurred at the far left on axis 1 of the CCA (Figure 2) and were only present at baits in forest (Figure 3; Table 1). Hooded crow (*Corvus cornix*) and white‐tailed eagle (*Haliaeetus albicilla*) occurred at baits in both habitats, but to a higher degree in forest. Red fox, common raven (*Corvus corax*), and golden eagle (*Aquila chrysaetos*) were clustered close to the midpoint of axis 1 (Figure 2) and occurred at bait stations in both habitats (Figure 3; Table 1). Arctic fox (*Vulpes lagopus*) and wolverine (*Gulo gulo*), the two most cold adapted species, were positioned well into the alpine tundra habitat (Figures 2 and 3). Arctic foxes occurred only at baits in the alpine tundra, while wolverine occurred at baits in both habitats, however only at higher altitudes in forest (Figures 2 and 3).

**FIGURE 4:**
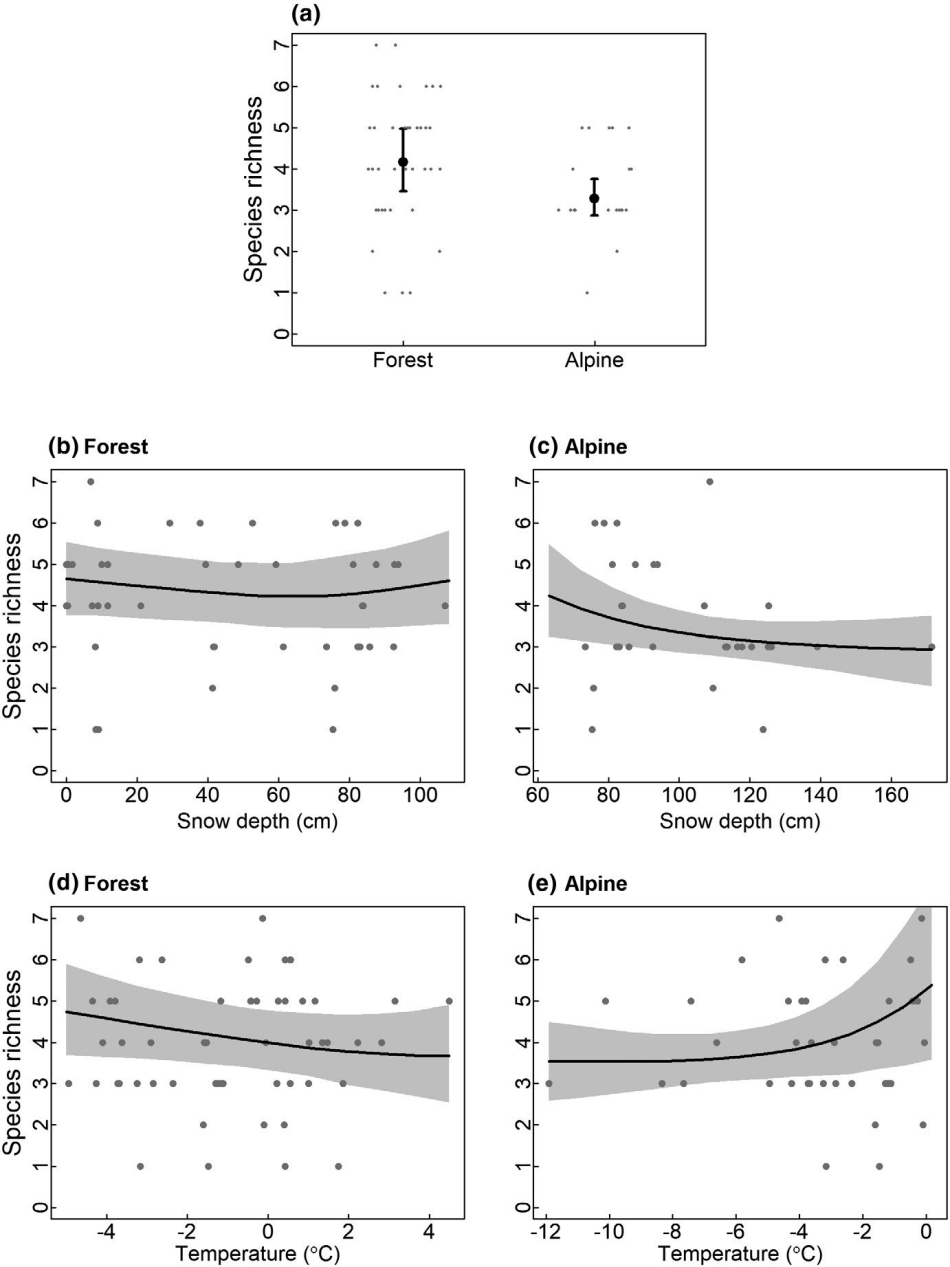
Results from the species community model, showing model predictions of species richness along environmental gradients. Shaded areas represent 95% credible intervals from the predicted posterior distributions, whereas points are observations at camera sites (bait sessions). (a) Habitat effect on species richness; (b, c) Effect of mean site snow depth on species richness in forested and alpine tundra habitats. (d, e) Effect of mean site temperature on species richness in forested and alpine habitats. Snow depth and temperature were constrained to their minimum/maximum values within each habitat type

**FIGURE 5:**
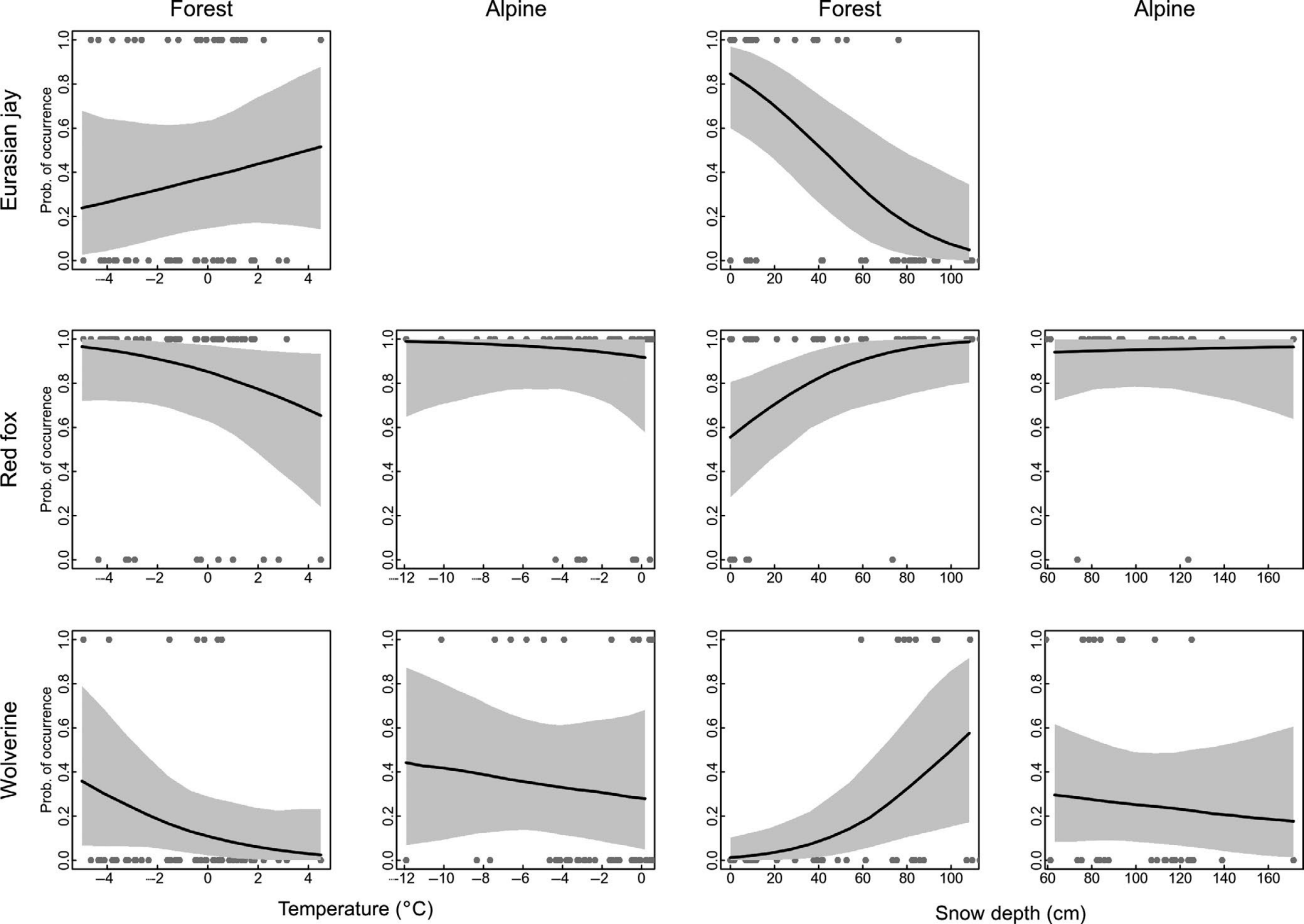
The probability of occurrence at baits in response to temperature and snow depth for selected species associated with either forest (Eurasian jay) or alpine tundra (wolverine), or habitat generalists (red fox). Solid lines are the predicted posterior probability of occurrence at baits from the species community model, whereas shaded gray areas represent 95% credible intervals. Gray points represent the bait sessions. Snow depth and temperature were constrained to their minimum/maximum values within each habitat type

The species community model showed a tendency for higher richness of scavenging species in forest than in alpine tundra habitats, with species‐specific associations to different habitats, similar to the CCA analysis (Figures 3 and 4a; Figure S4). Species richness was independent of snow depth in forested habitats (Figure 4b), whereas it declined with increasing snow depth in alpine tundra habitats (Figure 4c). Species richness decreased slightly with increasing temperature in forested habitats (Figure 4d) but increased slightly with warmer temperatures within alpine tundra habitats (Figure 4e). The impact of snow depth and temperature differed among species. Among corvids occurring at baits solely in forests (Table 1), only the Eurasian jay exhibited marked effects of these factors, that is, occurrence at baits was negatively affected by increasing snow depth and positively affected by increasing temperature (Figure 5). Most of the species occurring at baits in both habitats exhibited similar impacts of snow and temperature (common raven, golden eagle, red fox, wolverine). In general, neither of the two factors affected species occurrence at baits in alpine areas, but occurrence at baits in forest increased with increasing snow depth and declined with increasing temperature (Figure 5). We observed a different pattern in the occurrence at baits by hooded crow, which was negatively associated with snow depth and positively related to temperature at baits in alpine tundra. Weaker, but somewhat similar impacts of snow and temperature, were observed at baits in forest for this species (Figures S5a,b and S6a,b).

## 4 DISCUSSION

Although the occurrence at baits by different species changed along the forest–alpine gradient, we found a surprisingly small difference in species richness between forest and alpine tundra habitats. This relates to the fact that species richness, which we expressed as the number of different species visiting bait stations, is inherently a product of the probabilities of occurrence at baits by each of the different species present in each of the habitats. These probabilities are influenced by multiple factors that we will discuss below. However, the main reason behind the small difference in species richness between habitats likely relates to the high dominance of generalist species, such as common raven, golden eagle, and red fox that occur at baits along the whole gradient, and the low occurrence of several habitat specialists at baits.

The impacts of snow depth and temperature on species richness were weak in both forest and alpine habitats, but diverse effects were observed when examining species separately. Temperature and snow depth had a negligible effect on the occurrence at baits by species common in alpine tundra habitats, and consequently, species richness remained almost unaffected by the climatic variables in alpine tundra habitats. On the contrary, the impacts of temperature and snow depth on species were both stronger and more diverse in forest habitats. Opposite effects among different species in the forest likely evened out differences and resulted in relatively stable species richness along the environmental gradients of temperature and snow depth.

The contrasting effects of the environmental covariates on species occurrence at baits were evident when comparing the group of habitat generalists to the group of forest dwelling species. These patterns may be driven by several factors. Snow depth and temperature might affect factors typically associated with carrion use, such as the likelihood to find and access the bait, and the availability of alternative food sources (Killengreen et al., 2011; Pardo‐Barquín et al., 2019). However, snow depth and temperature might also restrict species winter range, area use, and activity patterns (Kowalczyk et al., 2003; Pozzanghera et al., 2016; Rivrud et al., 2019).

Species only found in forest were most common at baits when temperature was high and snow depth low. This group were dominated by smaller species potentially more affected by cold stress and less adapted to scavenge frozen meat than larger species. However, snow depth was the factor explaining most of the variation in occurrence of these species (Figure S3). This pattern was evident for all the smaller corvid species, despite that these species have different adaptations to harsh winter conditions. It is therefore likely that their access to baits was restricted by increasing snow depth. The low impact from temperature might indicate that the distribution of these species is not limited by temperature within our study area. It is also important to acknowledge that the impact from temperature might be higher at natural carcasses, as these might be harder to handle for smaller species compared to baits. The scavenging probability of the pine marten was also negatively affected by snow depth. Pine martens are adapted to locate and dig for food beneath snow. However, their efficiency in capturing voles, one of their main prey, is reduced with increasing snow depth (Willebrand et al., 2017). We suggest that the pine marten might adapt to increased snow depths by adjusting area use toward areas with less snow where there is a sufficient gradient in elevation, and hence snow depth, within their home range. On the contrary, occurrence at baits by the larger habitat generalists in forests was highest at deep snow and low temperature. Under such conditions, accessibility to important subnivean prey like small rodents is typically reduced, while energetic demands may increase. Accordingly, high occurrence at baits might simply be explained by increased importance of carrion when snow depth increases. The importance of carrion may also explain the contrasting responses to temperature and snow depth comparing occurrence at baits for the same group of generalists in alpine tundra versus forest habitat. In alpine tundra habitat, snow depth and temperature had little impact on these species' occurrence at baits. However, with our study design we could not detect more short term responses to snow conditions (Richard et al., 2014). The relationship between increased carrion use under periods of low availability of live prey and harshness of winter has been demonstrated for the most important mammalian habitat generalist, the red fox (Jędrzejewski & Jędrzejewska, 1992; Killengreen et al., 2011; Needham et al., 2014).

Following forecasted climate changes, a release in climatic constraints could be expected with shorter winters (fewer days with permanents snow cover), and higher winter temperatures (Räisänen & Eklund, 2012). This will likely result in range expansion of boreal scavengers. For instance, Eurasian jays and badgers have expanded their range in Scandinavian boreal forests since the 20th century, possibly driven by climate warming (Elmhagen et al., 2015). Our results suggest that within forest habitats, changes in snow conditions will have higher impact on scavengers than changes in temperature. This might be important e.g. when predicting the scavenging community responses to future climate change. Winter temperatures are expected to rise, even more at higher latitudes. Snowfall patterns might on the other hand show regional patterns, and snowfall is expected to decline more at lower altitudes (Räisänen & Eklund, 2012), affecting winter ranges for species limited by snow cover (Rivrud et al., 2019).

As several recent studies have pointed out, resource subsidies, including carrion, could facilitate generalist scavenger establishment and increased abundance in tundra ecosystems (Gallant et al., 2020; Henden et al., 2014; Sokolov et al., 2016). Our results add support to earlier studies pointing to carrion as an important subsidy for generalist scavengers during harsh winter conditions (Jędrzejewski & Jędrzejewska, 1992; Pulliainen & Ollinmäki, 1996; Temple, 1974). It might, on the other hand, be reasonable to infer from our results that carrion is of less importance for habitat generalists when snow is shallow. Taking into account that ungulate carrion biomass during winter is estimated to be eight times higher in forest compared to alpine tundra areas in this region (Hagen, 2014), and that ungulates often concentrate in areas with less snow, low occurrence at baits under such conditions might be explained by carrion saturation (Gomo et al., 2017).

Our study provides a temporal and spatial snapshot of the scavenger guild along a forest–alpine gradient in Fennoscandia during winter. Many scavengers have significant ecosystem impacts, acting as important predators (Jahren et al., 2016; Pereira et al., 2014) or superior competitors (Bodey et al., 2009; Elmhagen et al., 2017). Climate change might have direct impact on some species, while others might be more affected by changes in carrion availability. Carrion availability from wild and domestic ungulates is to a great extent affected by management practices (Henden et al., 2014; Selås & Vik, 2006), possibly strengthening ecosystem disturbance caused by changes in climatic conditions (Ims et al., 2019). Management should take both factors into account, and we encourage further studies of the underlying mechanisms driving observed patterns of scavenger occurrence at baits, as these might be important to understand and predict ongoing ecosystem changes.

## CONFLICT OF INTEREST

The authors declare no conflicts of interest.

## AUTHOR CONTRIBUTION

**Gjermund Gomo:** Conceptualization (equal); Data curation (equal); Formal analysis (supporting); Funding acquisition (equal); Investigation (equal); Methodology (equal); Project administration (equal); Resources (supporting); Visualization (supporting); Writing‐original draft (lead); Writing‐review & editing (lead). **Lars Rød‐Eriksen:** Conceptualization (supporting); Data curation (equal); Formal analysis (lead); Investigation (equal); Methodology (equal); Visualization (lead); Writing‐original draft (supporting); Writing‐review & editing (lead). **Harry P. Andreassen:** Conceptualization (supporting); Methodology (equal). **Jenny Mattisson:** Conceptualization (supporting); Formal analysis (supporting); Methodology (equal); Resources (supporting); Supervision (equal); Writing‐original draft (supporting); Writing‐review & editing (supporting). **Morten Odden:** Conceptualization (supporting); Supervision (equal); Writing‐original draft (supporting); Writing‐review & editing (supporting). **Olivier Devineau:** Conceptualization (supporting); Formal analysis (supporting); Methodology (equal); Supervision (equal); Writing‐original draft (supporting); Writing‐review & editing (supporting). **Nina E. Eide:** Conceptualization (equal); Data curation (supporting); Funding acquisition (equal); Investigation (equal); Methodology (equal); Project administration (equal); Resources (lead); Supervision (equal); Writing‐original draft (supporting); Writing‐review & editing (supporting).

## Supporting information

Fig S1

Fig S2

Fig S3

Fig S4

Fig S5a

Fig S5b

Fig S6a

Fig S6b

Table S1

Appendix S1

## Data Availability

The analyzed data are available in the Dryad digital repository: https://doi.org/10.5061/dryad.gxd2547h3
