# Supplementary figures and images for: Scavenger community structure along an environmental gradient from boreal forest to alpine tundra in Scandinavia
Source: Ecol Evol. 2020 Sep 25;10(23):12860–9. doi: 10.1002/ece3.6834 (PMC7713988; doi:10.1002/ece3.6834)

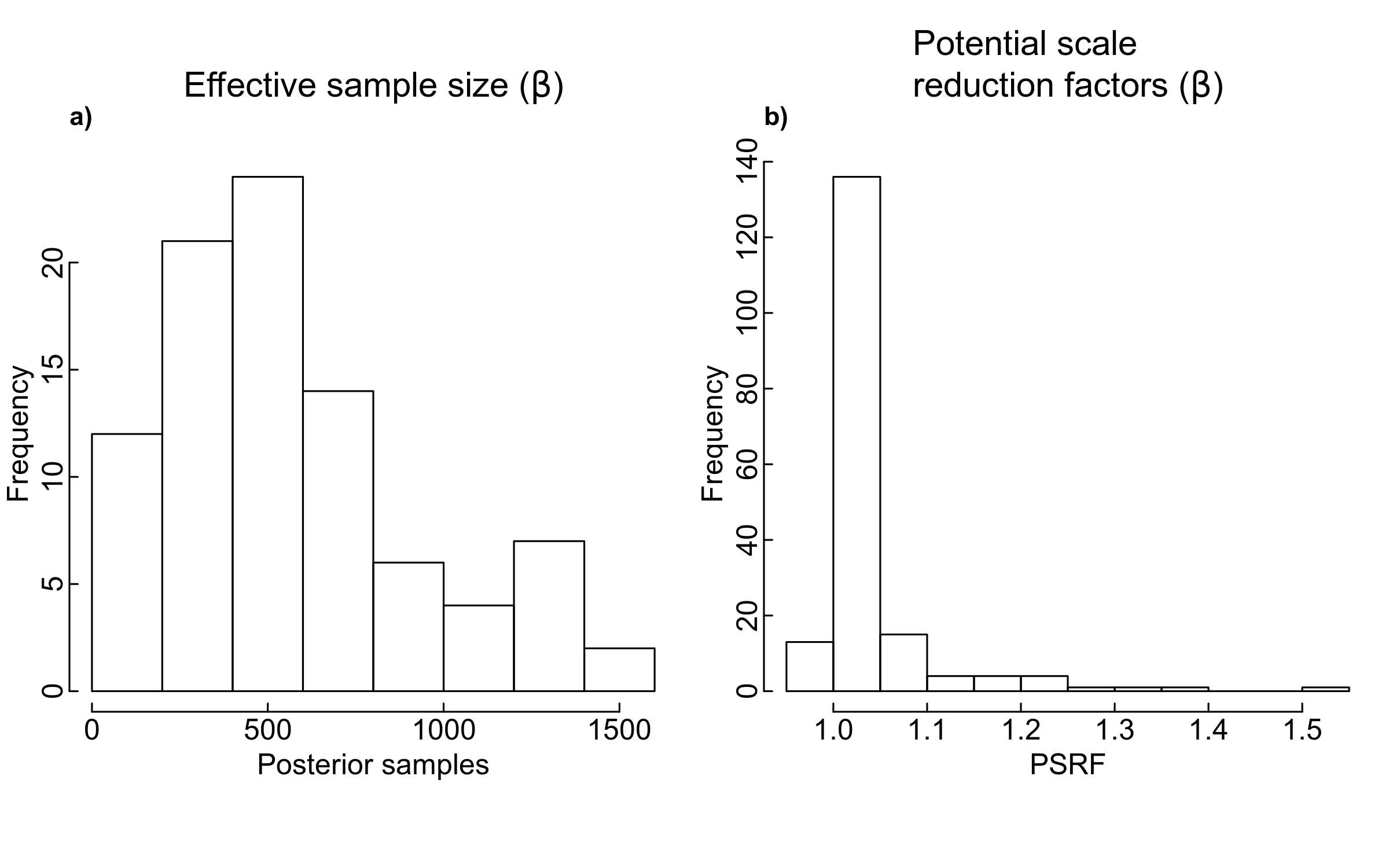

Supplement: Supplementary file 1 — Fig S1 [file ECE3-10-12860-s001.png]

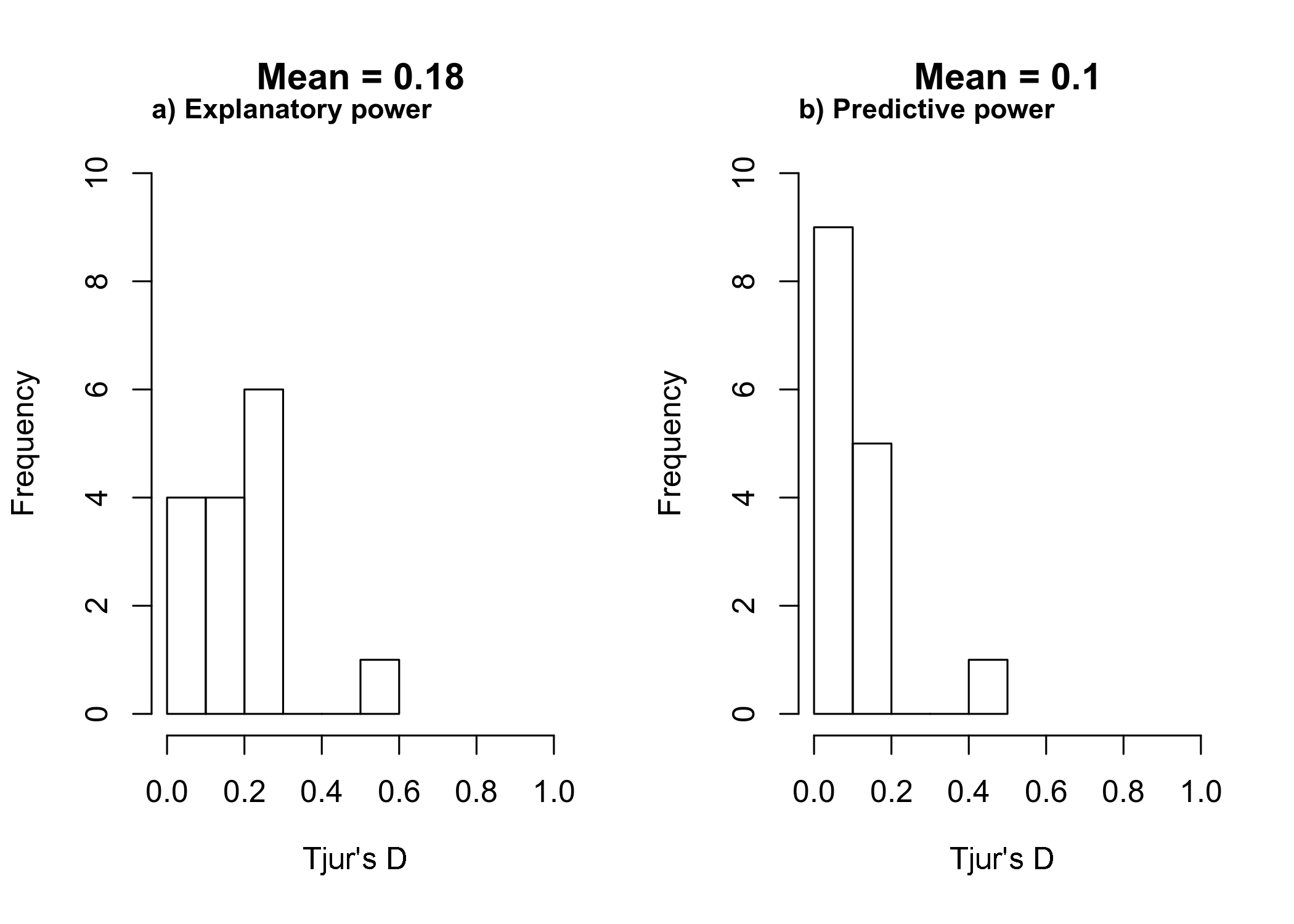

Supplement: Supplementary file 2 — Fig S2 [file ECE3-10-12860-s002.png]

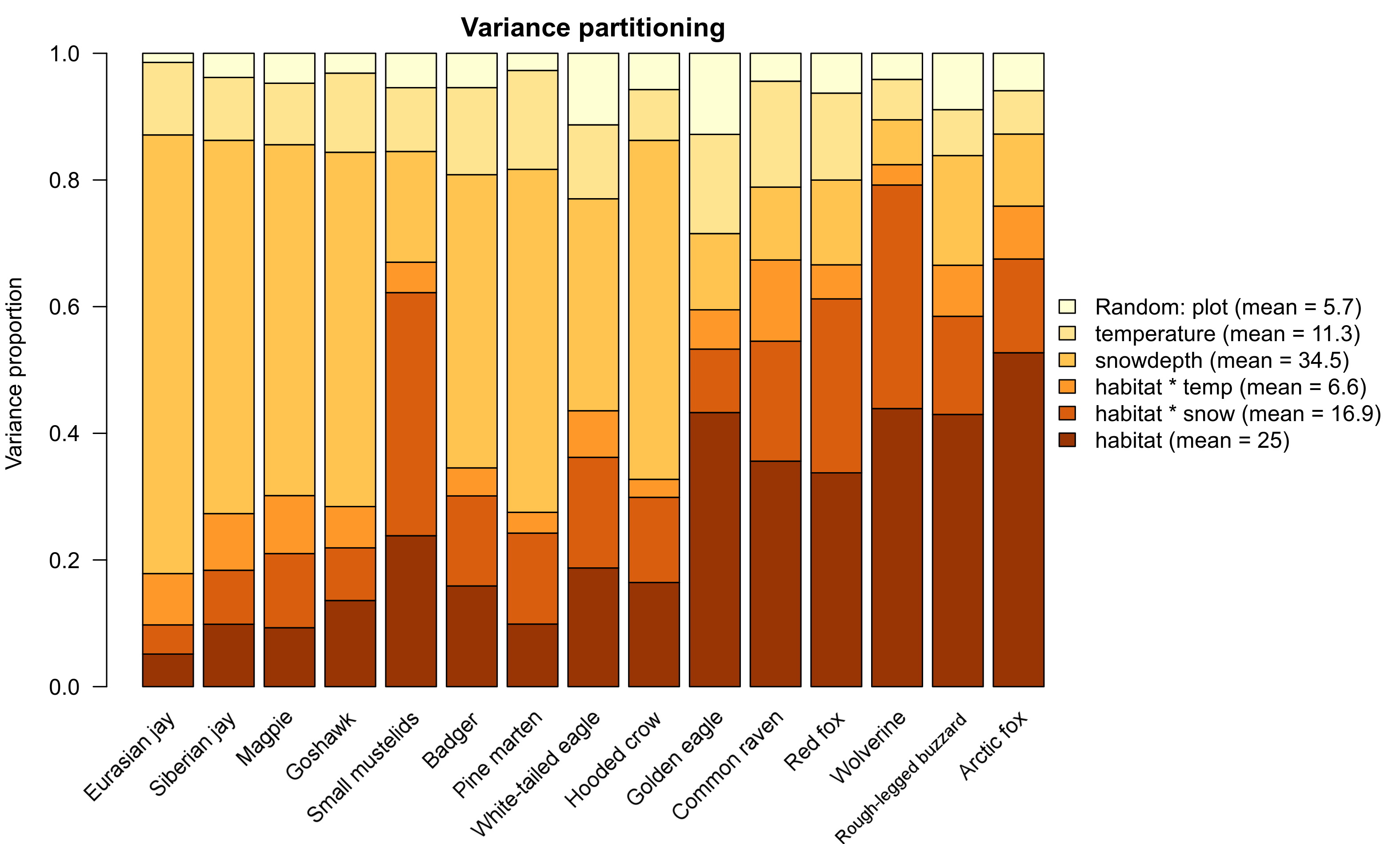

Supplement: Supplementary file 3 — Fig S3 [file ECE3-10-12860-s003.png]

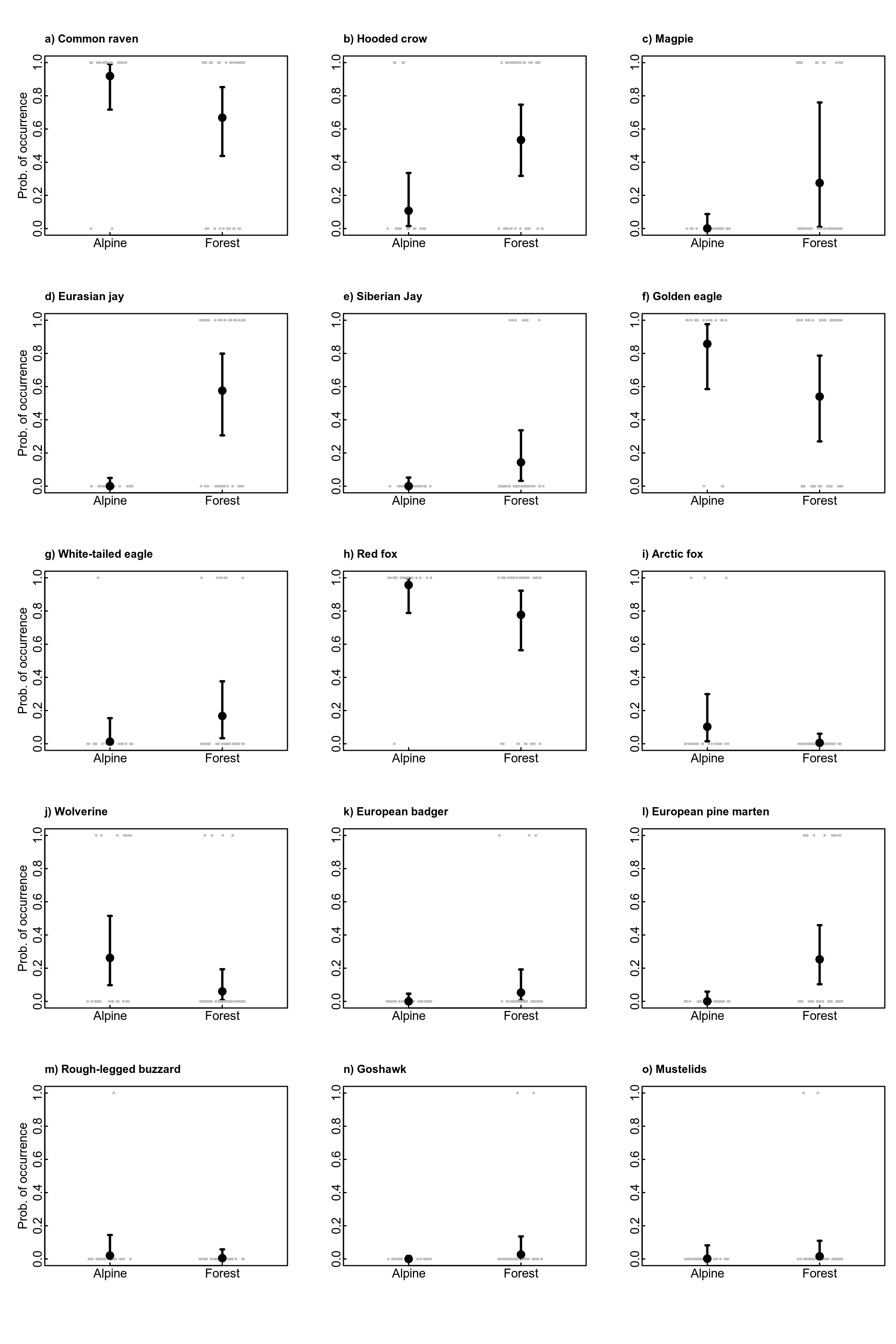

Supplement: Supplementary file 4 — Fig S4 [file ECE3-10-12860-s004.png]

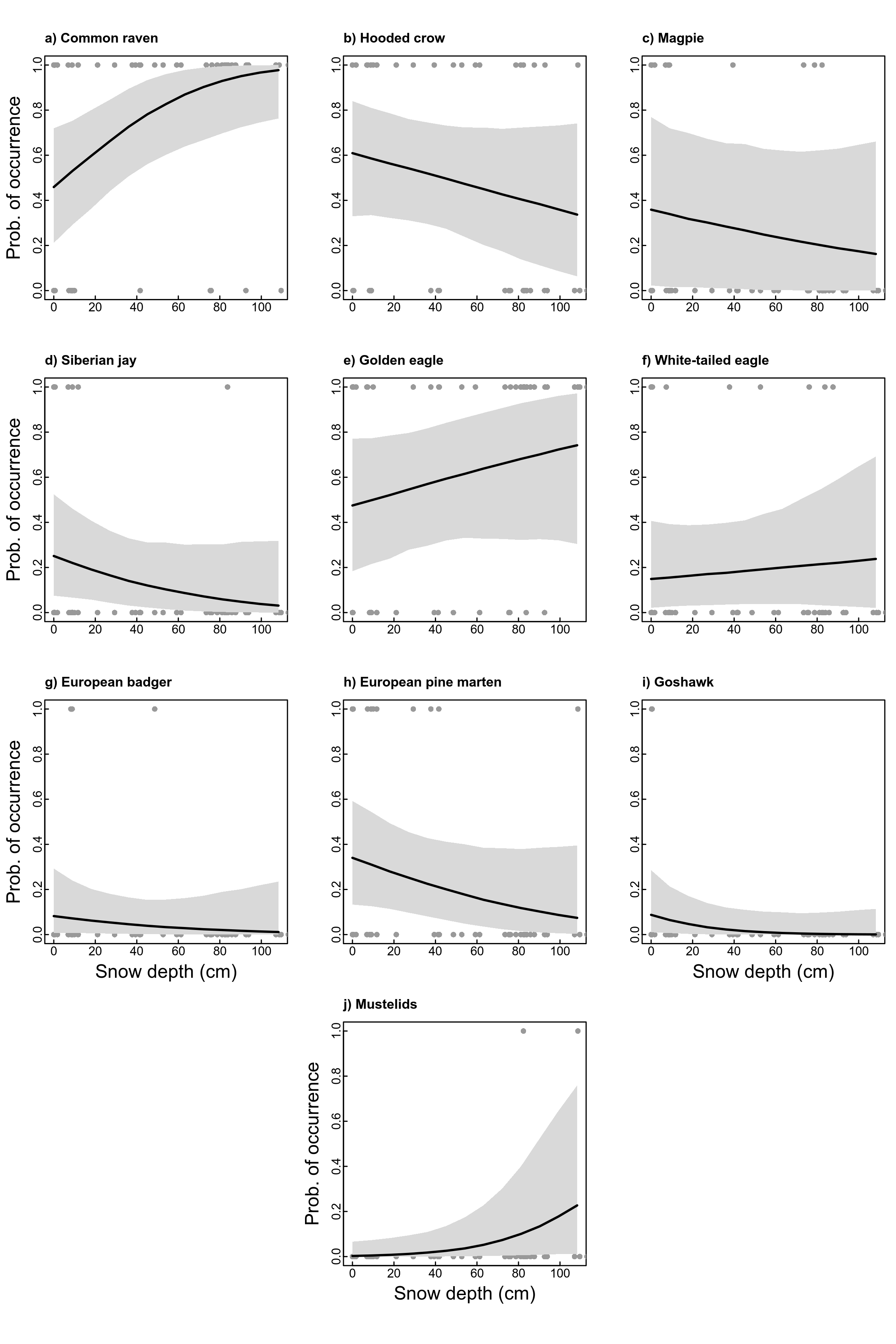

Supplement: Supplementary file 5 — Fig S5a [file ECE3-10-12860-s005.png]

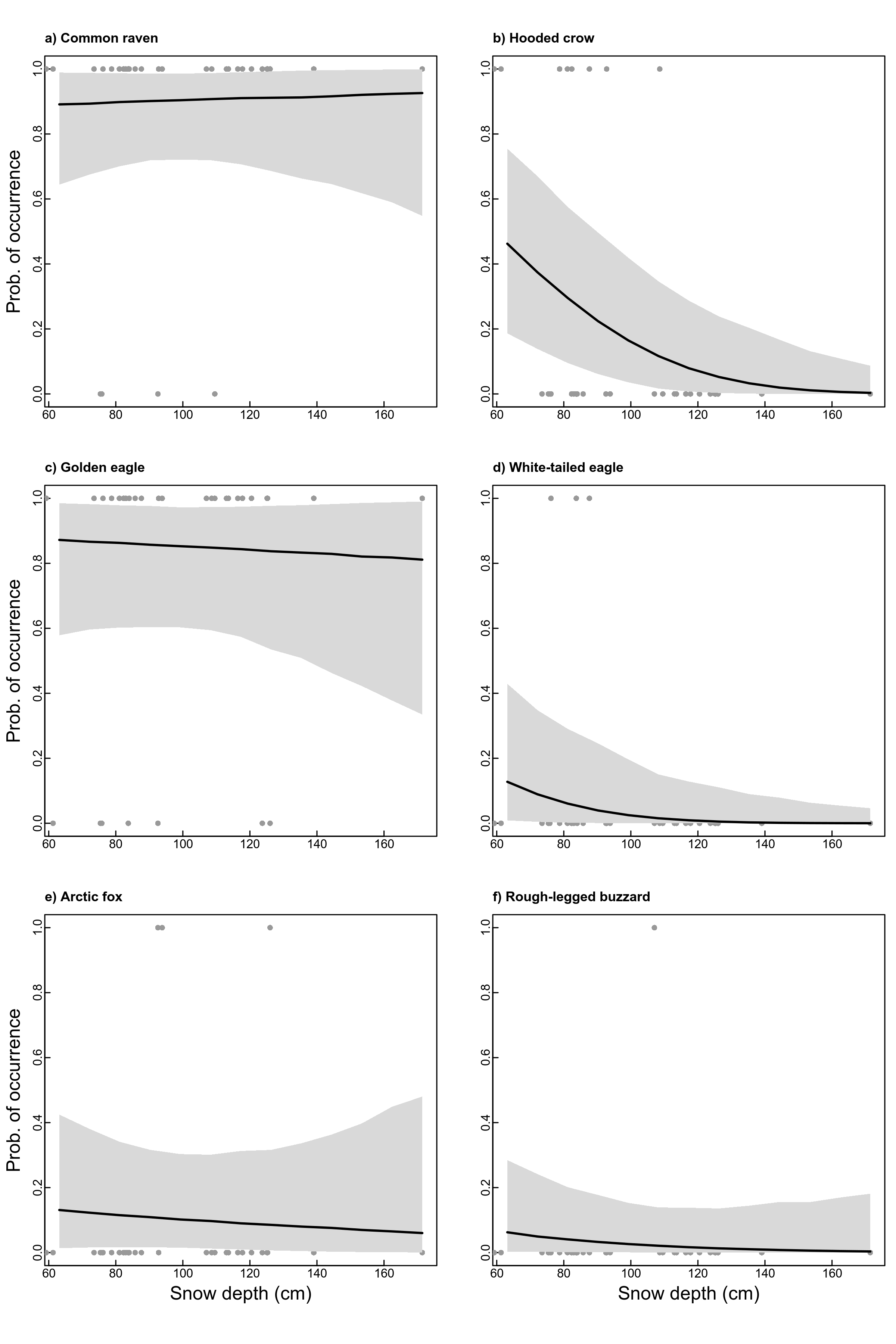

Supplement: Supplementary file 6 — Fig S5b [file ECE3-10-12860-s006.png]

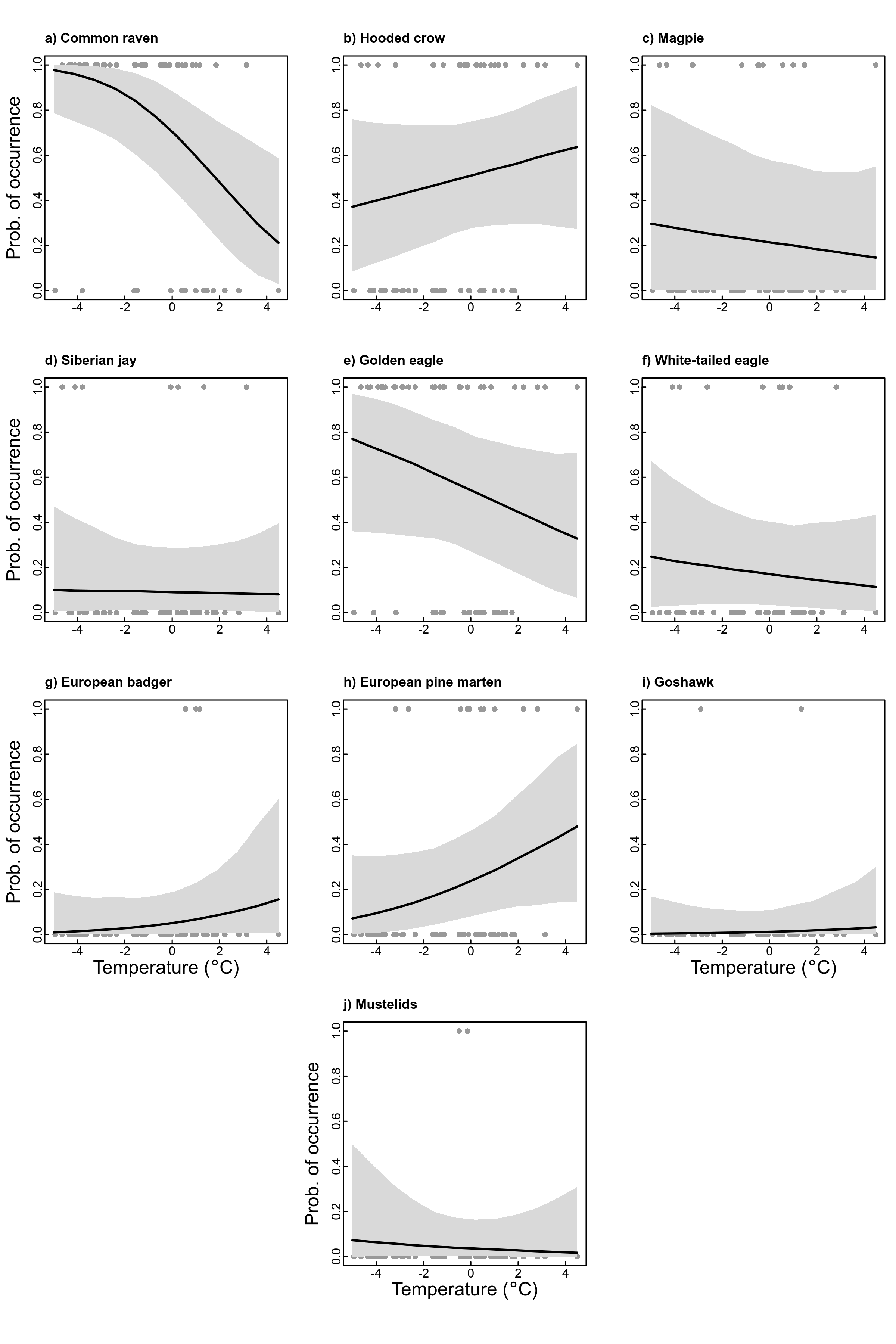

Supplement: Supplementary file 7 — Fig S6a [file ECE3-10-12860-s007.png]

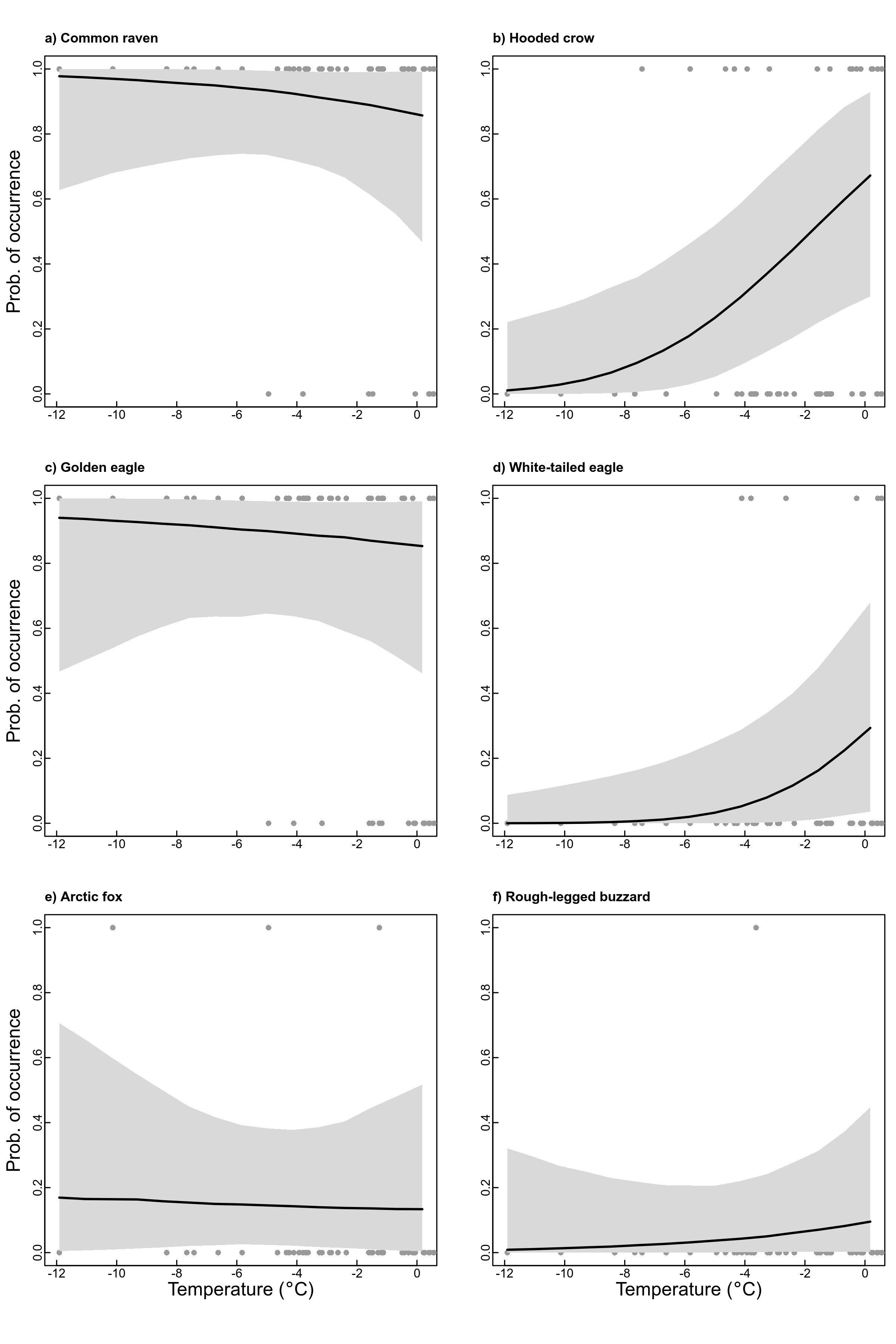

Supplement: Supplementary file 8 — Fig S6b [file ECE3-10-12860-s008.png]
