# Supplementary material for: Scavenger community structure along an environmental gradient from boreal forest to alpine tundra in Scandinavia
Source: Ecol Evol. 2020 Sep 25;10(23):12860–9. doi: 10.1002/ece3.6834 (PMC7713988; doi:10.1002/ece3.6834)
Supplement: Supplementary file 9 — Table S1 [file ECE3-10-12860-s009.docx]

**Table S1**. Results from the multivariate linear regression (JSDM) showing coefficient (β) estimates for each species and covariate. The table shows the numerical means on the probit link scale for each species and covariate, whereas numbers in brackets are the 95% credible interval (2.5%, 97.5%). Interaction terms (habitat * snow and habitat * temp) are for the forest habitat.

|  | **(Intercept)** | **Habitat: Forest** | **Snow depth** | **Temperature** | **Habitat*Snow.** | **Habitat*Temp.** |
| --- | --- | --- | --- | --- | --- | --- |
| Common raven | 1.057 [-0.452, 2.792] | -1.134 [-2.861, 0.413] | -0.001 [-0.155, 0.154] | -0.093 [-0.309, 0.084] | 0.130 [-0.065, 0.327] | -0.120 [-0.384, 0.149] |
| Hooded crow | 1.420 [0.140, 2.783] | -1.143 [-2.455, 0.133] | -0.216 [-0.366, -0.078] | 0.086 [-0.072, 0.258] | 0.163 [0.002, 0.332] | -0.046 [-0.268, 0.173] |
| Magpie | 0.577 [-1.155, 2.215] | -0.934 [-2.446, 0.837] | -0.302 [-0.566, -0.098] | 0.153 [-0.097, 0.533] | 0.204 [-0.027, 0.461] | -0.273 [-0.700, 0.024] |
| Eurasian jay | 1.590 [-0.264, 3.371] | -0.538 [-2.240, 1.424] | -0.409 [-0.705, -0.171] | 0.211 [-0.088, 0.680] | 0.126 [-0.147, 0.448] | -0.316 [-0.801, 0.037] |
| Siberian jay | 0.382 [-1.464, 1.977] | -1.069 [-2.668, 0.717] | -0.307 [-0.572, -0.095] | 0.160 [-0.121, 0.565] | 0.164 [-0.083, 0.445] | -0.268 [-0.691, 0.047] |
| Golden eagle | 1.175 [-0.248, 2.884] | -1.257 [-2.885, 0.161] | -0.040 [-0.193, 0.106] | -0.080 [-0.286, 0.080] | 0.079 [-0.082, 0.256] | -0.022 [-0.248, 0.225] |
| White-tailed eagle | 0.246 [-1.278, 1.727] | -1.303 [-2.724, 0.144] | -0.188 [-0.375, -0.029] | 0.151 [-0.059, 0.430] | 0.202 [0.016, 0.404] | -0.188 [-0.505, 0.087] |
| Red fox | 1.336 [-0.366, 3.274] | -1.191 [-3.155, 0.498] | 0.005 [-0.163, 0.170] | -0.085 [-0.339, 0.114] | 0.187 [-0.014, 0.416] | 0.064 [-0.197, 0.355] |
| Arctic fox | -0.865 [-2.812, 0.825] | -2.012 [-3.747, -0.421] | -0.057 [-0.224, 0.111] | -0.053 [-0.205, 0.098] | 0.138 [-0.113, 0.381] | 0.066 [-0.286, 0.443] |
| Wolverine | -0.346 [-1.770, 1.209] | -1.955 [-3.586, -0.645] | -0.057 [-0.203, 0.071] | -0.078 [-0.211, 0.055] | 0.276 [0.100, 0.484] | 0.056 [-0.190, 0.328] |
| European badger | -0.065 [-1.931, 1.604] | -1.327 [-2.919, 0.433] | -0.263 [-0.568, -0.051] | 0.175 [-0.099, 0.534] | 0.216 [-0.044, 0.511] | -0.066 [-0.459, 0.297] |
| European pine marten | 0.552 [-1.310, 2.314] | -0.989 [-2.635, 0.908] | -0.295 [-0.561, -0.090] | 0.196 [-0.073, 0.560] | 0.232 [-0.001, 0.487] | -0.081 [-0.462, 0.236] |
| Rough-legged buzzard | -0.828 [-2.901, 0.875] | -1.899 [-3.614, -0.303] | -0.101 [-0.302, 0.088] | 0.034 [-0.168, 0.271] | 0.156 [-0.111, 0.405] | -0.007 [-0.369, 0.379] |
| Goshawk | -0.078 [-2.430, 1.769] | -1.296 [-3.136, 0.963] | -0.324 [-0.674, -0.068] | 0.180 [-0.134, 0.750] | 0.140 [-0.184, 0.477] | -0.220 [-0.792, 0.161] |
| Small mustelids | -1.171 [-3.637, 0.784] | -1.722 [-3.569, -0.004] | -0.148 [-0.390, 0.076] | 0.112 [-0.170, 0.469] | 0.363 [0.121, 0.671] | -0.031 [-0.457, 0.369] |
