# Supplementary material for: Scavenger community structure along an environmental gradient from boreal forest to alpine tundra in Scandinavia
Source: Ecol Evol. 2020 Sep 25;10(23):12860–9. doi: 10.1002/ece3.6834 (PMC7713988; doi:10.1002/ece3.6834)
Supplement: Supplementary file 10 — Appendix S1 [file ECE3-10-12860-s010.docx]

**Supporting information**

**Table S2**. Results from the CCA model (ANOVA: F_3,57_ = 4.071; p < 0.001) including habitat (factorial, forested and alpine), temperature and snow depth (continuous). Continuous variables consist of yearly (i.e. within study period) means for each site. The left table shows statistics of included terms, whereas the right table shows statistics by CCA axis, from an ANOVA analysis.

| *Parameter* | *df* | *X^2^* | *F-statistic* | *p-value* |  | *Axis* | *df* | *Χ^2^* | *F-statistic* | *p-value* |
| --- | --- | --- | --- | --- | --- | --- | --- | --- | --- | --- |
| habitat | 1 | 0.179 | 7.460 | < 0.001 |  | CCA1 | 1 | 0.265 | 11.041 | < 0.001 |
| temperature | 1 | 0.036 | 1.479 | 0.141 |  | CCA2 | 1 | 0.019 | 0.781 | 0.935 |
| snow depth | 1 | 0.079 | 3.273 | < 0.001 |  | CCA3 | 1 | 0.010 | 0.390 | 0.967 |
| Residual | 57 | 1.367 |  |  |  | Residual | 57 | 1.367 |  |  |


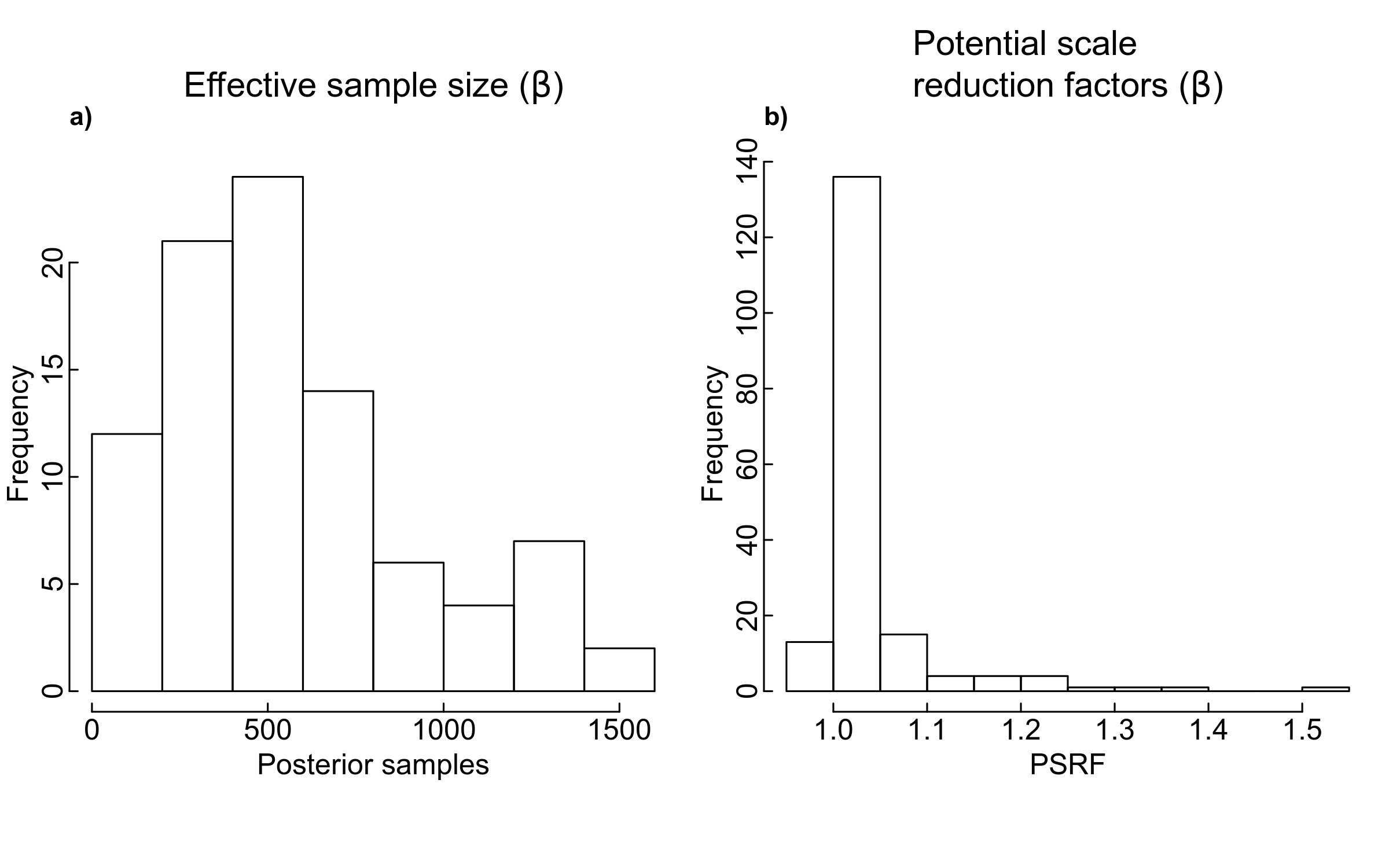


**Figure S1**. Assessment of convergence of the species community model. **a**) the distribution of the effective posterior sample size of βparameters (optimal = 2000). **b**) the distribution of the potential scale reduction factors of β parameters (optimal = 1.00).


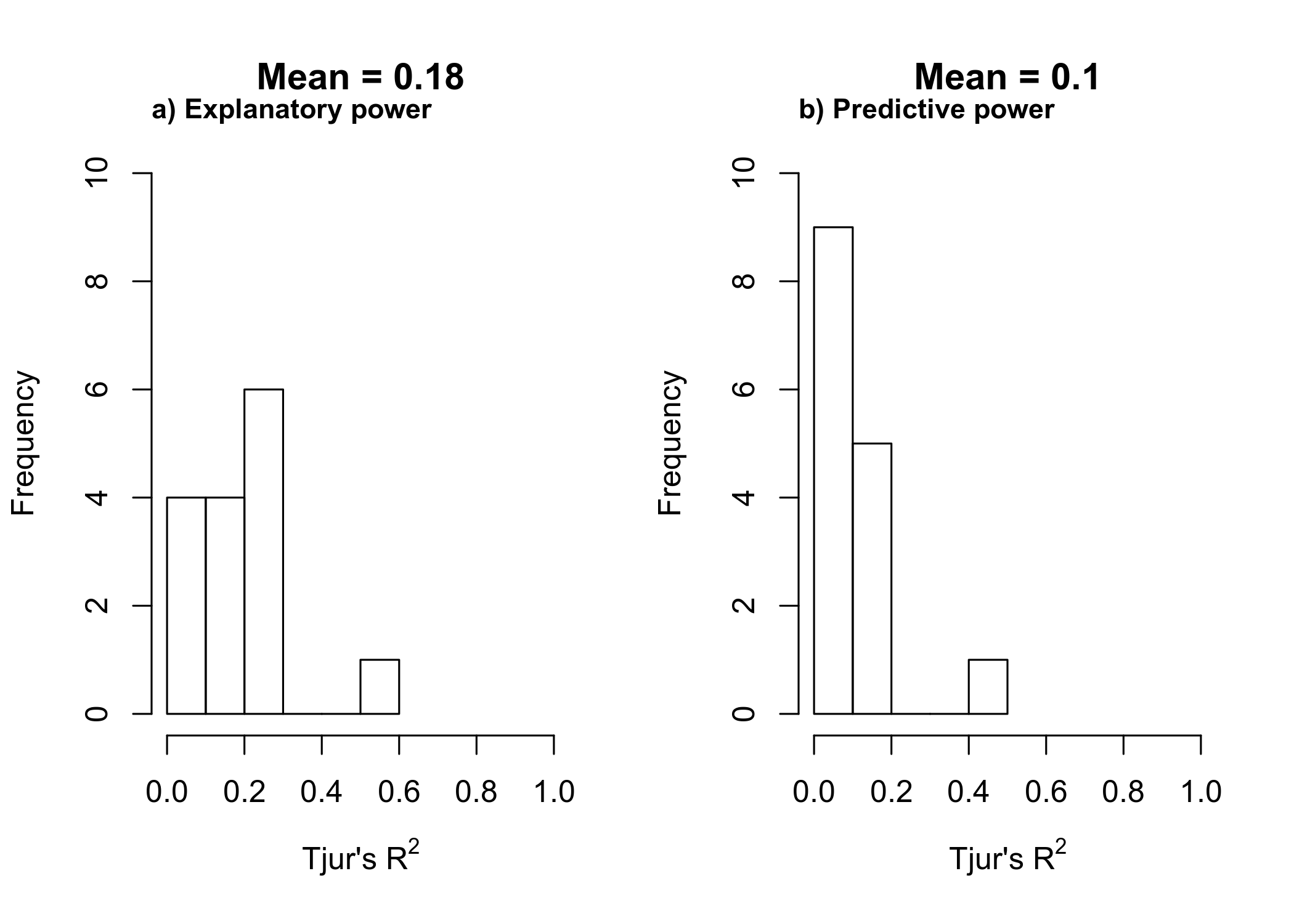


**Figure S2**. Model validation using Tjur’s coefficient of discrimination (R^2^) for logistic models. **a**) the proportion of variance explained by the model for each species. **b**) the predictive power of the model for each species, obtained from a 5-fold cross validation.


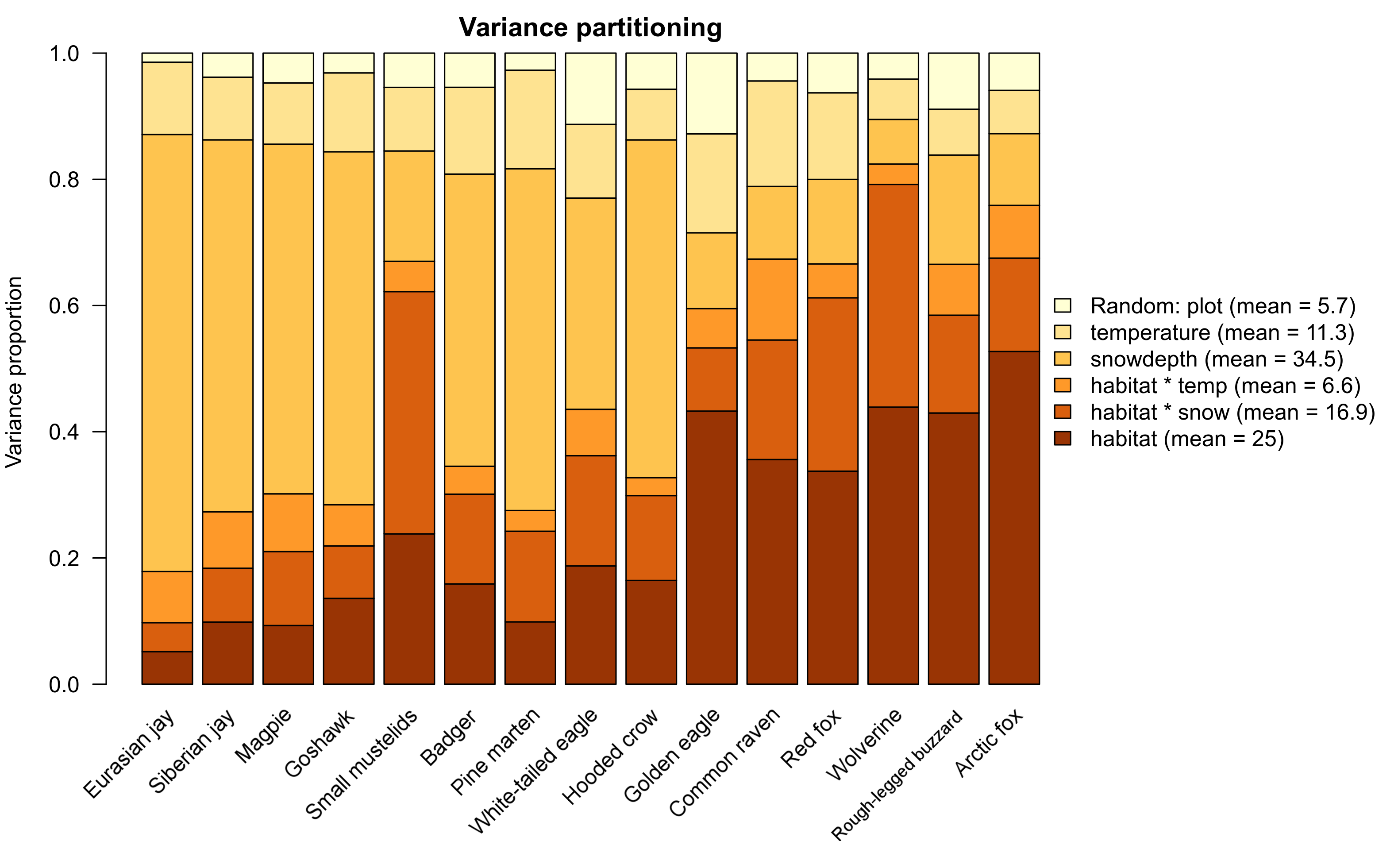
**Figure S3**. Variance partitioning of the species community model, showing to which degree occurrence at baits by each species is explained by explanatory variables. Random indicates leftover unexplained variance for each species relative to the other species.


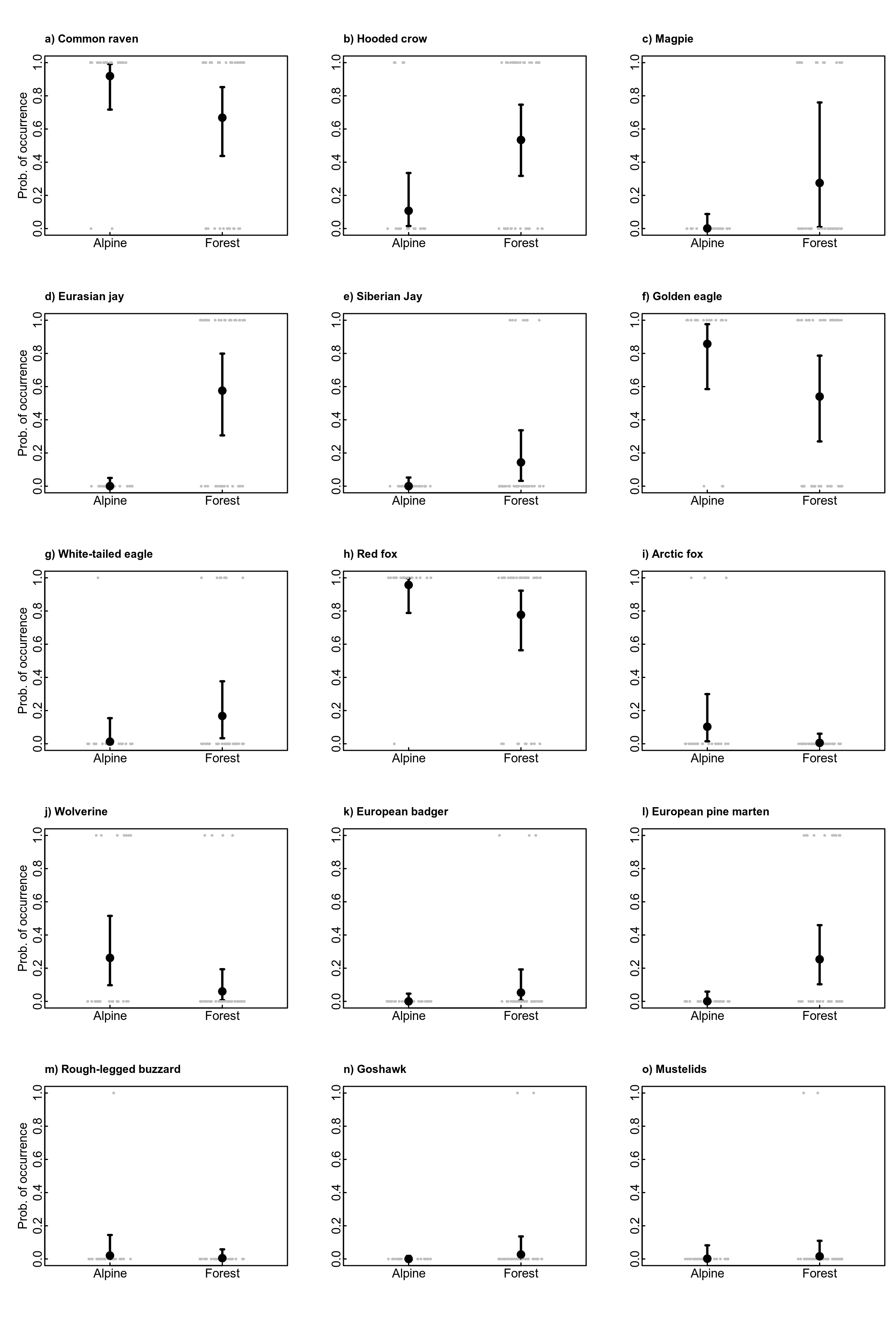


**Figure S4**. Habitat effects on the probability of species occurrences at baits, predicted from the species community model. Black points are the predicted probability of occurrence at baits, whereas vertical bars represent 95% credible intervals. Grey points are the camera stations (bait sessions).


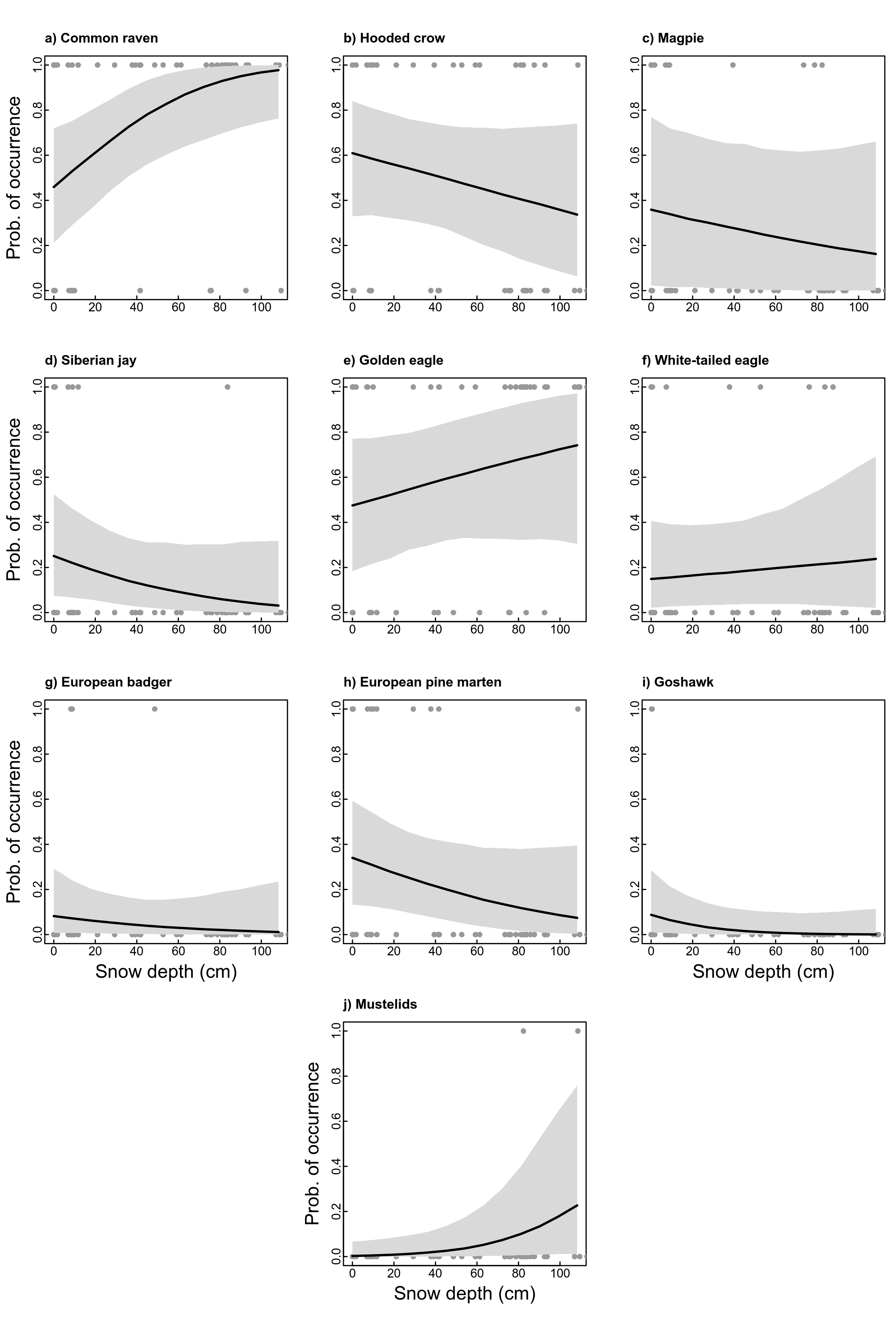


**Figure S5a**. Effects of snow depth (cm) on the probability of species occurrences at baits within forest habitats, predicted from the species community model. Shaded areas represent 95% credible intervals, whereas grey points are the camera stations (bait sessions). Plotted effects are constrained to the minimum/maximum value of snow depth within forested habitats. Only species occurring at baits within forested habitats are presented.


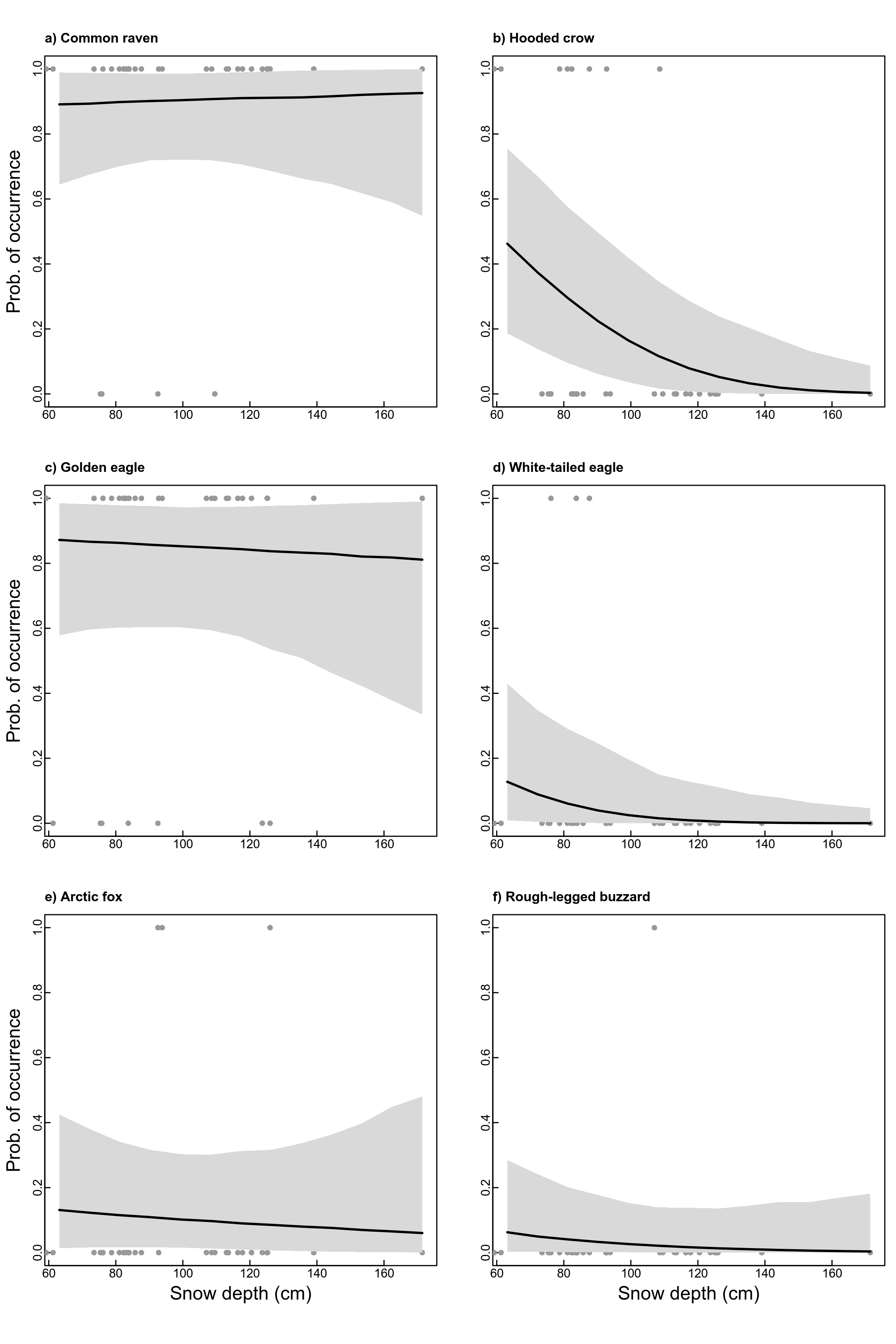


**Figure S5b.** Effects of snow depth (cm) on the probability of species occurrences at baits within alpine habitats, predicted from the species community model. Shaded areas represent 95% credible intervals, whereas grey points are the camera stations (bait sessions). Plotted effects are constrained to the minimum/maximum value of snow depth within alpine habitats. Only species occurring at baits within alpine tundra habitats are presented.


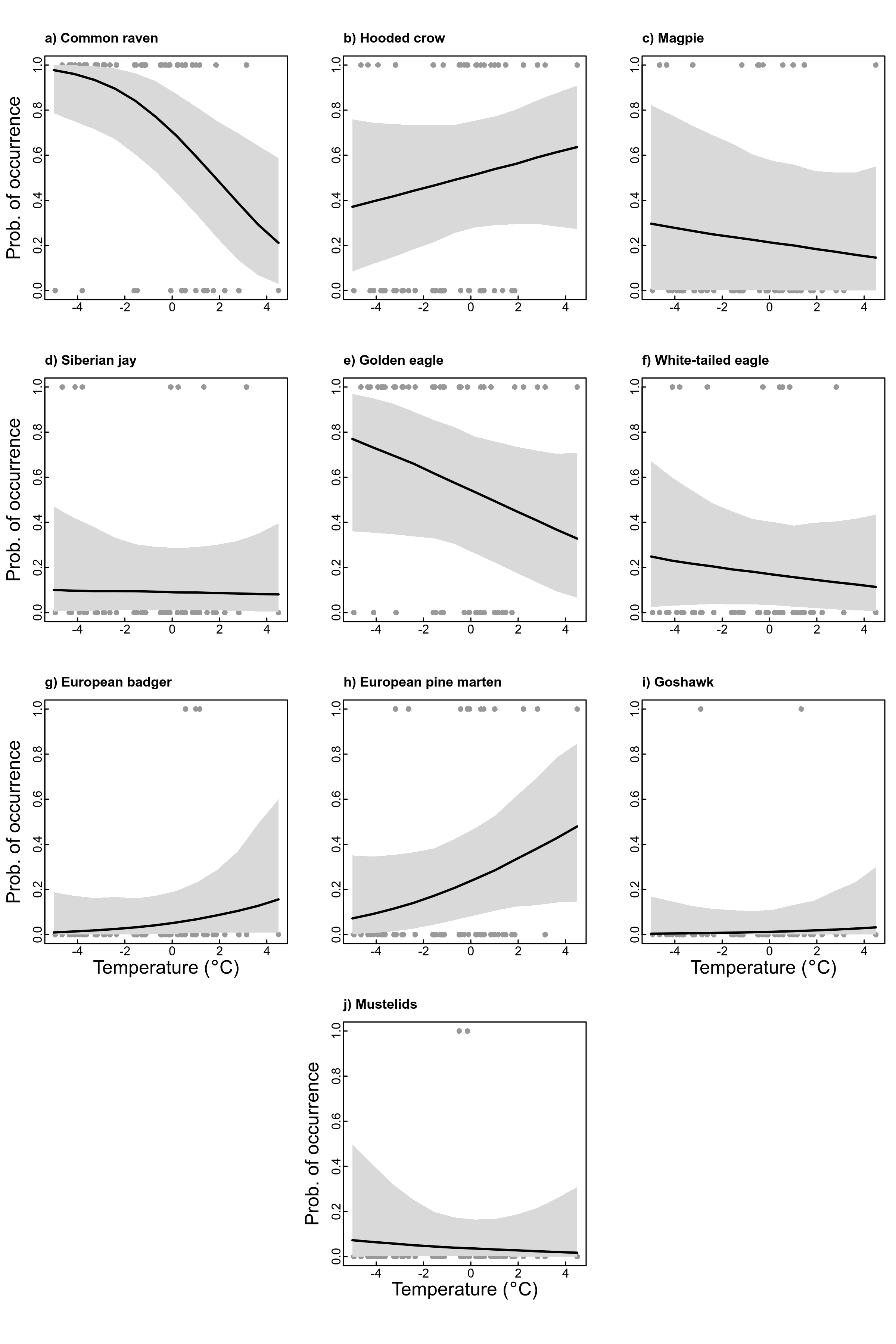


**Figure S6a**. Effects of temperature (°C) on the probability of species occurrences at baits within forested habitats, predicted from the species community model. Shaded areas represent 95% credible intervals, whereas points are the camera stations (bait sessions). Plotted effects are constrained to the minimum/maximum value of temperature within forested habitats. Only species occurring at baits within forested habitats are presented.


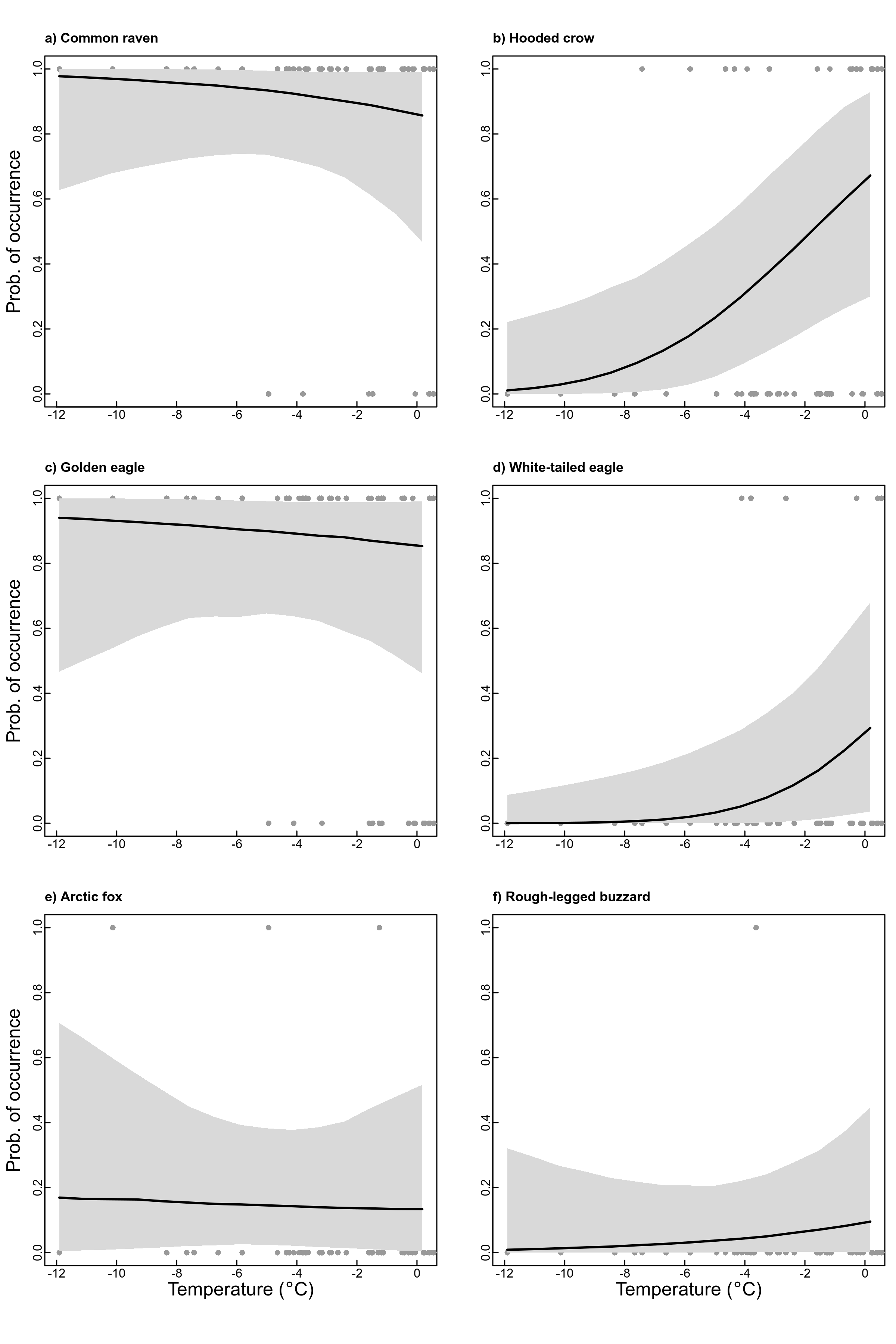


**Figure S6b**. Effects of temperature (°C) on the probability of species occurrences at baits within alpine habitats, predicted from the species community model. Shaded areas represent 95% credible intervals, whereas points are the camera stations (bait sessions). Plotted effects are constrained to the minimum/maximum value of temperature within alpine habitats. Only wspecies occurring at baits within alpine tundra habitats are presented.


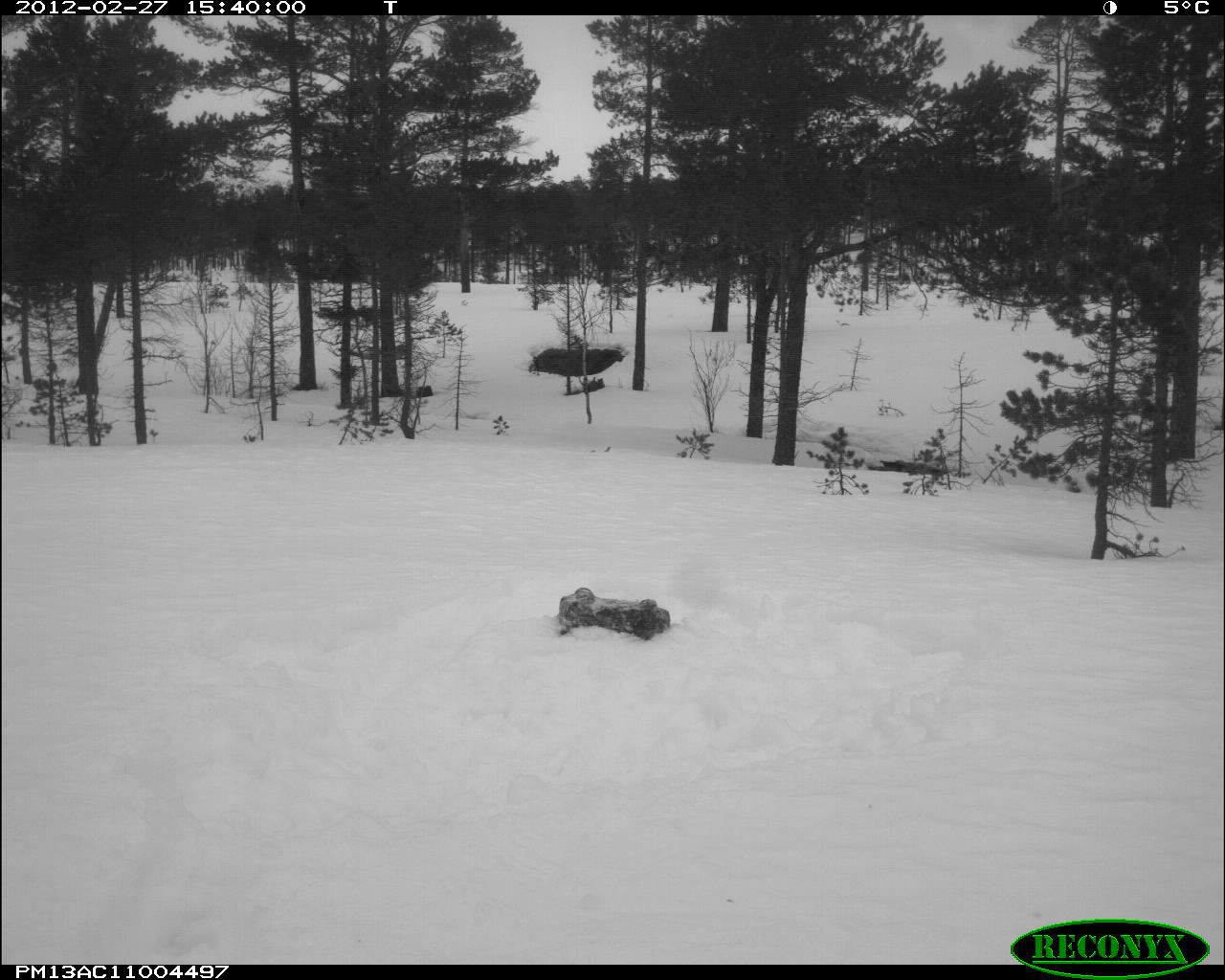


**Figure S7a**. Example of first image of bait after setup.


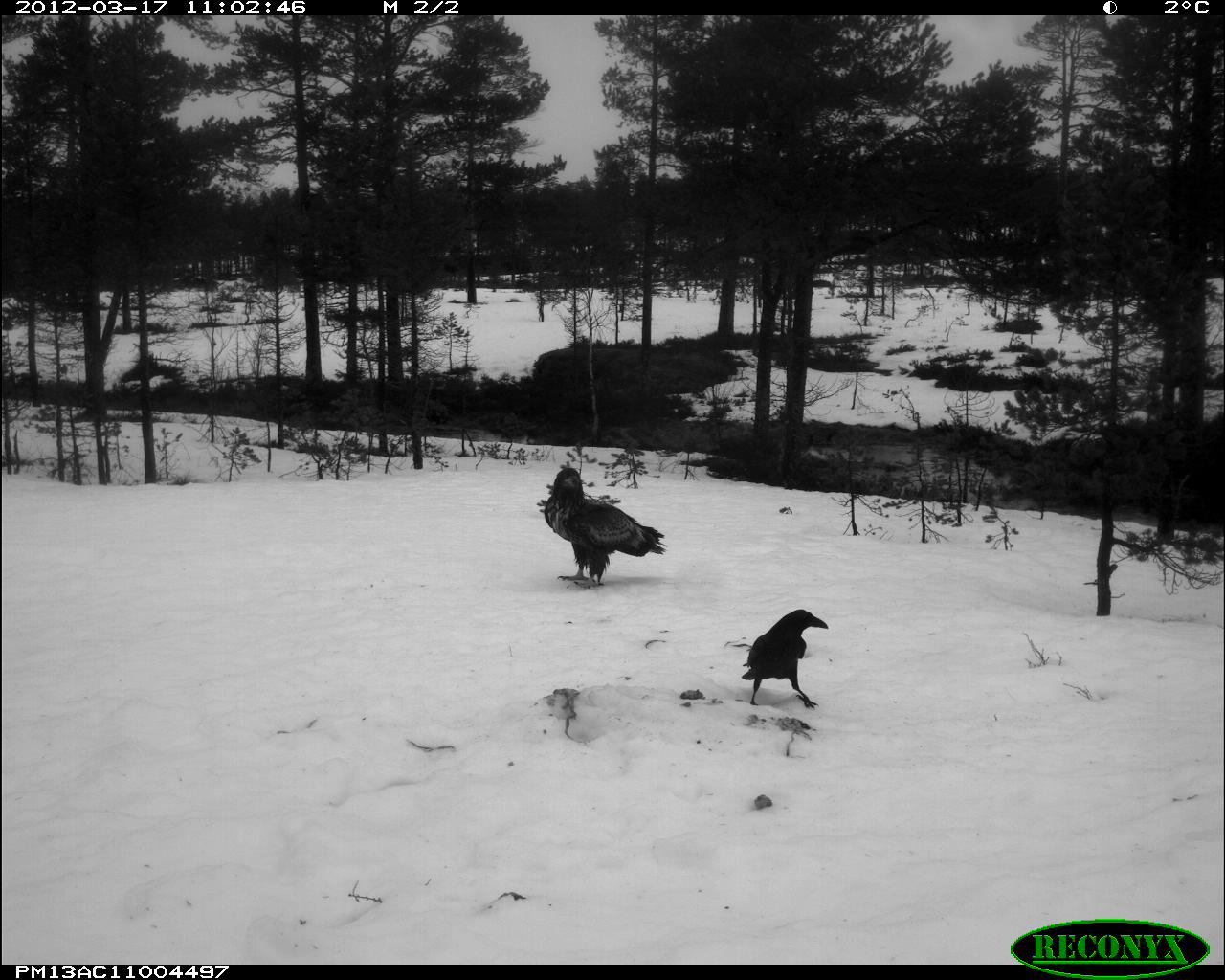


**Figure S7b**. Example of near depleted bait included in the analyses.


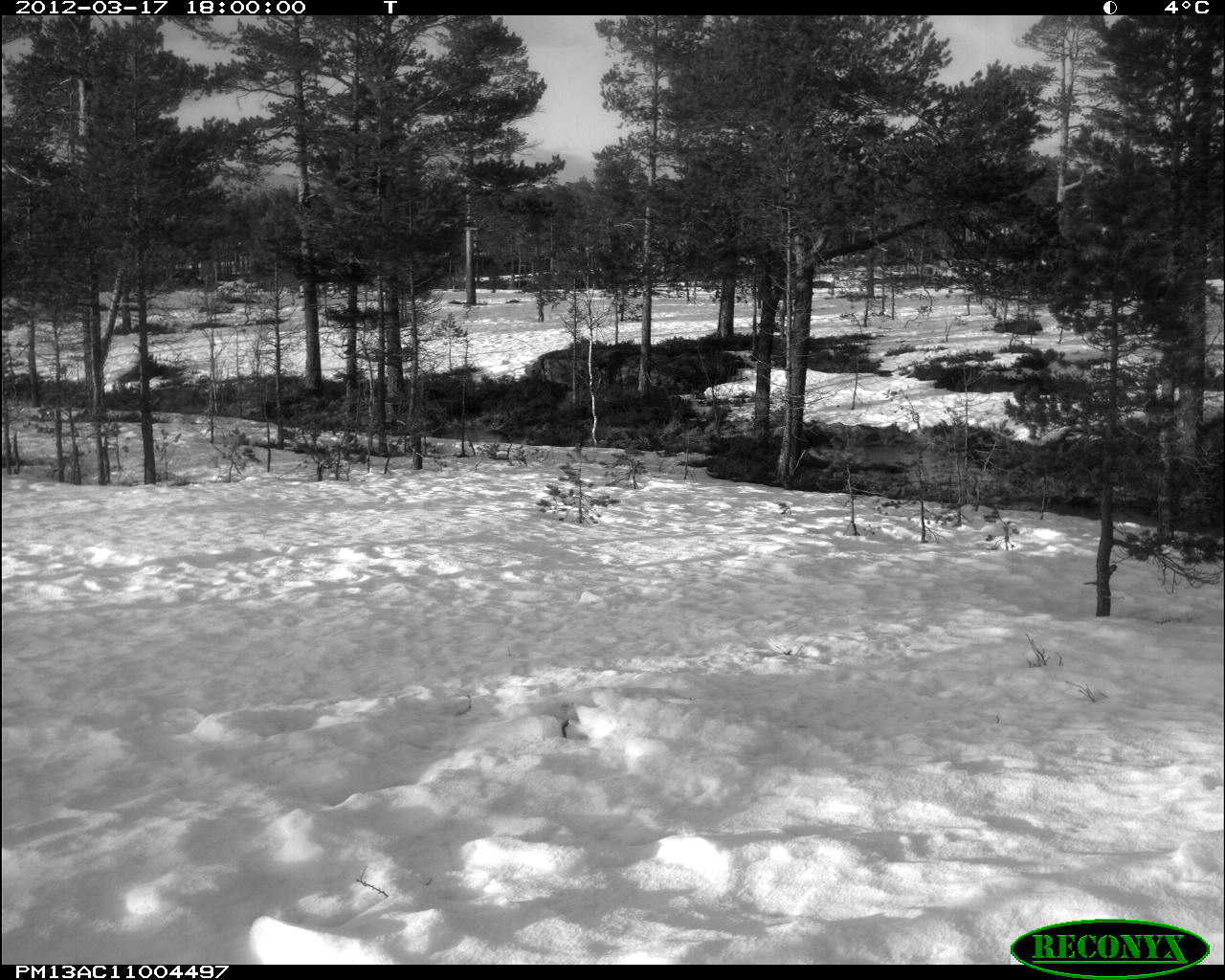


Figure S7c. Example of image of depleted bait, not included in the analyses.
